# Correlated variability in primate superior colliculus depends on functional class

**DOI:** 10.1038/s42003-023-04912-0

**Published:** 2023-05-18

**Authors:** Leor N. Katz, Gongchen Yu, James P. Herman, Richard J. Krauzlis

**Affiliations:** 1 Laboratory of Sensorimotor Research, National Eye Institute, Bethesda, MD 20892 USA; 2 Department of Ophthalmology, University of Pittsburgh, Pittsburgh, PA 15219 USA

**Keywords:** Neural decoding, Perception

## Abstract

Correlated variability in neuronal activity (spike count correlations, r_SC_) can constrain how information is read out from populations of neurons. Traditionally, r_SC_ is reported as a single value summarizing a brain area. However, single values, like summary statistics, stand to obscure underlying features of the constituent elements. We predict that in brain areas containing distinct neuronal subpopulations, different subpopulations will exhibit distinct levels of r_SC_ that are not captured by the population r_SC_. We tested this idea in macaque superior colliculus (SC), a structure containing several functional classes (i.e., subpopulations) of neurons. We found that during saccade tasks, different functional classes exhibited differing degrees of r_SC_. “Delay class” neurons displayed the highest r_SC_, especially during saccades that relied on working memory. Such dependence of r_SC_ on functional class and cognitive demand underscores the importance of taking functional subpopulations into account when attempting to model or infer population coding principles.

## Abstract (web summary)

Electrophysiological recordings in the macaque superior colliculus reveal that correlated variability (r_SC_) depends on functional class.

## Introduction

Neuronal responses to similar sensorimotor settings are variable, and often correlated between neurons of a population. Such correlated variability (spike-count correlations, r_SC_) is important in systems neuroscience because the degree of r_SC_ can have profound implications on population codes^1–4^. When making judgements about sensory events, perceptual accuracy can be dramatically impaired if the shared variability in neuronal responses mimics the stimulus-driven activity^5–7^. Therefore, identifying the factors that determine r_SC_ during behavioral tasks is critical for understanding how neurons interact to guide behavior^8,9^.

What are the factors that determine r_SC_? Mechanistically, the presence of r_SC_ in a population is evidence of common inputs onto the neurons. The origin of such inputs, however, is still debated^2,10,11^. Seminal experiments^1,12^ had attributed the presence of r_SC_ to shared noise in the sensory inputs, which led to the term “noise correlation” in reference to r_SC_^2,5,6^. Today, however, the term “noise correlations” is largely appreciated as a misnomer as the shared variability is driven less by noise on sensory inputs and instead, by signals that had yet to be identified at the time. For example, r_SC_ can be driven by global fluctuations in population activity that are not noise^13–16^, and by task-related feedback signals that target specific neuronal populations^11,17,18^. Consistent with the idea that r_SC_ is largely due to feedback signals, changes in r_SC_ have been observed following a variety of experimental manipulations aimed at influencing cognitive states such as attention^19,20^, perceptual learning^21,22^, decision-making^8,11^, and task context^11,19^.

We reasoned that separate sources of input may carry separate signals that differ in their degree of r_SC_, such that separate subpopulations of neurons would exhibit different degrees of r_SC_ that are related to their function, processing stage or association with either feedforward or feedback projections. Despite the expectation that r_SC_ would vary across subpopulations of neurons within a population, most reports of r_SC_ in the literature are summarized as a single value per population of neurons (or per brain area)^6^. Such an approach is useful insofar as the summary value reflects the constituent elements of the population. However, if the population consists of subpopulations with distinct levels of r_SC_, a single-value summary would obscure such distinctions in the data and could misinform models of information transfer in the brain.

We tested whether different subpopulations of a population exhibit different levels of r_SC_ in the primate superior colliculus (SC), because SC neurons are readily classified into distinct subpopulations (or functional classes) based on their spiking response properties during saccade tasks. Neurons defined as either visual, visual-movement, movement, or delay correspond to different locations within the SC circuitry and are linked to distinct sources of input and output^23–26^. We computed the degree of r_SC_ within and between functional classes over multiple epochs of two saccade tasks and found that different classes of neurons exhibited distinct levels of r_SC_ that were not accurately captured by the overall population r_SC._ Neurons belonging to the “Delay” class displayed the highest correlation values amongst all classes, and these were particularly high during epochs that involved working memory. “Movement” class neurons, in contrast, exhibited the lowest level of r_SC,_ even during the saccadic eye movement. Such variability in r_SC_ and its dependence on cognitive demand indicates that subpopulations of neurons occupy distinct niches within the SC network, and likely receive distinct modulatory inputs from upstream regions. We conclude that the circuit niche of subpopulations of neurons, at least as reflected by their functional class, is important to consider to accurately inform population coding models in SC, or elsewhere in the brain.

## Results

**Fig. 1:**
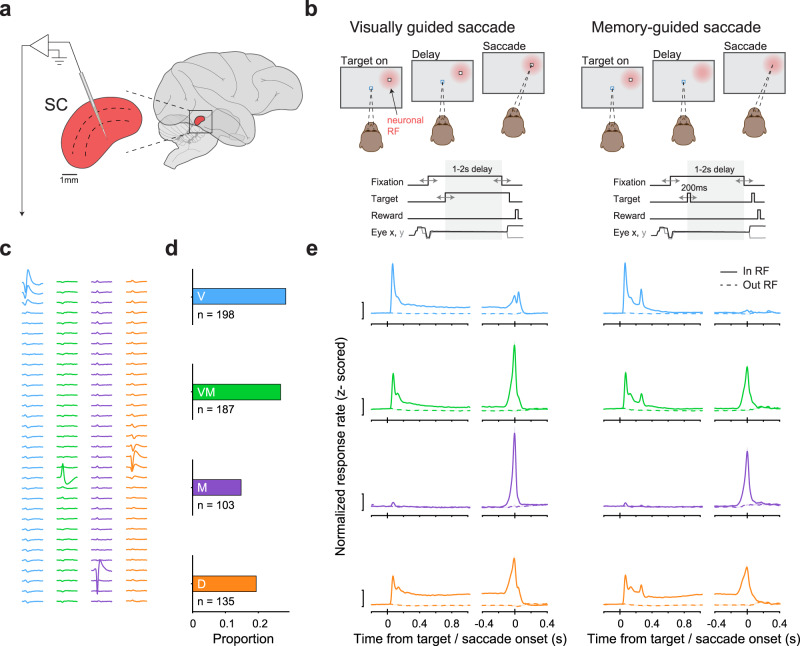
SC neurons were readily classified into functional classes during a guided saccade task. **a** A multichannel probe was positioned to primarily target the intermediate layers of SC in macaques performing a guided saccade task. **b** Monkeys performed either visually guided (left) or memory-guided saccades (right). Top: in either task condition, the subject was required to maintain fixation during both target presentation and a 1–2 s delay period. The offset of the fixation point cued the monkey to saccade to the target to obtain a juice reward. The key difference between task conditions is that the target did not remain visible during the delay period of memory-guided saccade trials. Visually guided and memory-guided saccade trials were interleaved, and the target could appear within the envelope of neuronal receptive fields (RF, indicated by the red patch) or elsewhere. Bottom: timing of task events. **c** Example waveforms on each probe channel (rows) from isolated neurons from a single recording session, one set from each functional class (colors matched to panels **d** and **e**). **d** Proportion of neurons recorded from each functional class: Visual (V); Visual-Movement (VM); Movement (M); and Delay (D). **e** Population average of *z*-score normalized responses for each of the classes for saccades into the neuronal RF (“in RF”) and out (“out RF”), relative to target onset and saccade onset, during visually guided (left) and memory-guided saccade trials (right). Scale bar equals a *z*-score of 1. Error bars are 1 SEM, bootstrapped, shaded, and often occluded by the mean. The *n* for each class is indicated in panel d.

We recorded from the superior colliculus (SC) of two nonhuman primates (*Macaca mulatta*) trained to perform saccadic eye movements. During each recording session, monkeys performed visually guided and memory-guided saccades on separate, randomly interleaved trials (Fig. 1a, b). Whereas the saccade target remained visible during the delay period of the visually guided saccade condition, it was absent from the memory-guided version, requiring the monkey to rely on their working memory of target location to guide the saccade. Saccade target location on each trial was selected randomly from among multiple locations, including a location within the envelope of neuronal receptive fields (RF), termed the “in-RF” target. Only successfully completed trials (74%) were used for analysis (see Methods).

A multichannel recording probe was positioned to span multiple layers of the SC such that groups of neurons with different functional properties were recorded simultaneously on each recording session (Fig. 1a, c). Overall, we recorded 699 neurons with well-defined RFs over 45 recording sessions (Monkey R: 314 neurons over 16 sessions; Monkey P: 385 neurons over 29 sessions). The results presented below were quantitatively consistent across the two monkeys and were therefore combined.

### SC neurons were readily classified into functional classes

SC neurons were classified into distinct functional classes following well-established criteria, based on their responses during the visual, delay, and movement periods of the memory-guided saccade task (see Methods). Applying statistical criteria used previously^27^, we assigned each neuron to a functional class: “Visual” (28%); “Visual-Movement” (27%); “Movement” (15%); and neurons exhibiting persistent activity during the delay period of the memory-guided saccade task, termed “Delay” neurons (19%) (Fig. 1d). The mean response of each subpopulation reflected their assigned class (especially pronounced in the memory-guided saccade task, the right column of Fig. 1e) and was consistent with previous reports^27–29^ in both saccade tasks (Fig. 1e).

### Correlated variability in SC depended on functional class

The classification of SC neurons into subpopulations enabled us to test whether different classes of SC neurons exhibited different degrees of correlated variability, and how these vary with task demands. For neurons within a hemisphere, r_SC_ was only computed for neuron pairs with overlapping RFs, netting 4626 pairs of simultaneously recorded neurons. Within each functional class, our dataset included 477, 470, 104, and 269 pairs from the Visual, Visual-Movement, Movement, and Delay classes, respectively. Across functional classes, our dataset included an additional 3306 pairs.

**Fig. 2:**
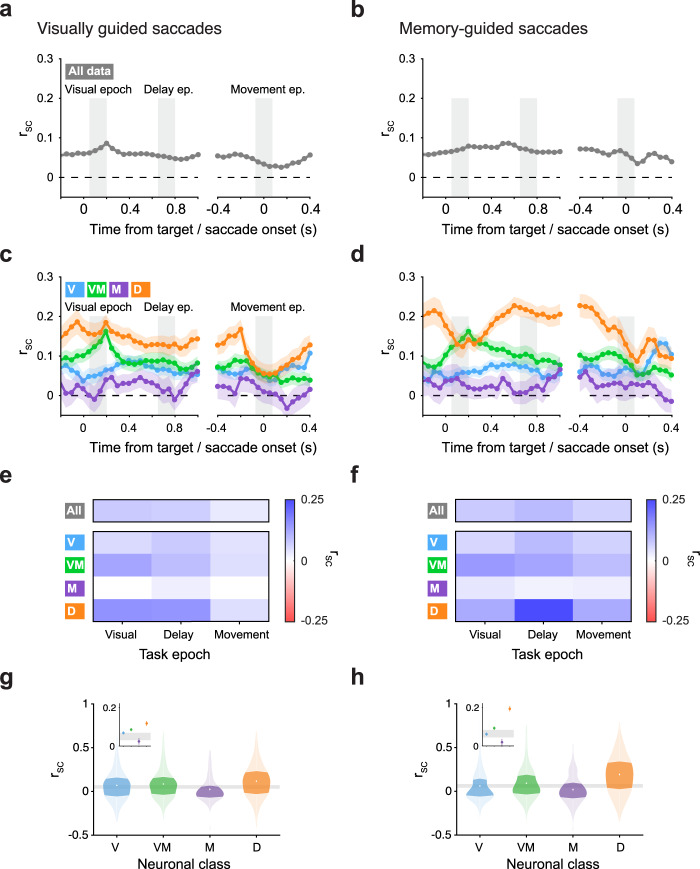
r_SC_ in SC depended on functional class. **a**, **b** Time course of correlated variability (r_SC_) measured across all SC neurons collapsed across class (*n* pairs = 4626) during visually guided (**a**) and memory-guided (**b**) saccade trials, relative to target and saccade onset (150 ms bins in 50 ms steps). Three key epochs used for subsequent analysis are indicated: visual epoch (50–200 ms after target onset); delay epoch (500–1000 ms after target onset); and movement epoch (75 ms before to 75 ms after saccade onset). **c**, **d** Time course of r_SC_ measured for distinct subpopulations of SC neuron pairs, classified as either Visual (V; *n* pairs = 477), Visual-Movement (VM; *n* pairs = 470), Movement (M; *n* pairs = 104), or Delay (D; *n* pairs = 269). Same format as (**a**, **b**). **e**, **f** Top bar: Mean r_SC_ in each epoch for SC neuron pairs collapsed across class during visually guided (**e**) and memory-guided (**f**) saccade trials. Bottom matrix: Mean r_SC_ in each epoch for SC neuron pairs from each functional class. **g**, **h** Violin plots show the distribution of r_SC_ values for each functional class during the delay epoch of visually guided (**g**) and memory-guided (**h**) saccades. Darker regions indicate the interquartile range. Gray horizontal bar reflects 95% confidence intervals on the mean of r_SC_ values for pairs drawn from a class-blind “null” distribution, bootstrapped. Insets zoom-in on functional classes’ means with 1 SEM error bars, bootstrapped.

As an initial characterization of correlated variability among SC neurons, we measured the time course of spike-count correlations during both types of saccade tasks, computed over all SC neuron pairs regardless of functional class (Fig. 2a, b). Partitioning SC neuron pairs into distinct functional classes revealed time courses of r_SC_ that varied substantially across neuronal class (Fig. 2c, d), and were strikingly different from the r_SC_ computed on all SC pairs independent of class (Fig. 2a, b). To statistically quantify how r_SC_ values varied across classes we considered r_SC_ values in the three key task epochs indicated on Fig. 2a–d: the visual epoch, the delay epoch, and the movement epoch (see “Methods” and shaded regions on Fig. 2a–d). We found that for visually guided saccades (Fig. 2e), the degree of r_SC_ depended strongly on task epoch, on neuronal class, and on the interaction between them (all *P* < 0.001, two-way ANOVA). The highest values of r_SC_ were exhibited by neurons from the Visual-Movement and Delay classes, predominantly during the visual and delay epochs of the task. The lowest value of r_SC_ was displayed by the Movement class neurons, even during the movement epoch itself. For saccades that rely on working memory (Fig. 2f), a similar pattern was observed with significant effects of task epoch, neuronal class, and the interaction between them (all *P* < 0.001, two-way ANOVA). One conspicuous difference between visually and memory-guided saccades, however, is the higher level of Delay class r_SC_ during the delay epoch of memory-guided saccades, an effect we consider more directly later.

The dependence of r_SC_ on functional class was most striking during the delay epoch. Targeted analysis of this epoch revealed clear differences in r_SC_ across classes (Fig. 2g, h). Mean r_SC_ values during visually guided saccade trials were positive in each neuronal class individually (Fig. 2g) and significantly different from a matched trial-shuffled (null) distribution (Supplementary Fig. 1a). Moreover, r_SC_ values across classes significantly differed from one another (*P* < 0.001, ANOVA). Similar results were obtained for memory-guided saccade trials; neuron pairs in each of the four classes exhibited mean r_SC_ values that were significantly larger than zero (Fig. 2h and Supplementary Fig. 1b) and significantly different from one another (*P* < 0.001, ANOVA), but with a much larger difference between pairs in the Delay class and in all other classes.

Thus, neurons that belong to distinct functional classes exhibited differing degrees of r_SC_, and these depended on task epoch and on whether working memory was required to perform the task. Spike-count correlations were highest in Delay class neurons, and this was especially pronounced during the delay epoch of memory-guided saccades.

### The dependence of r_SC_ on functional class was not due to firing rate, distance, signal correlation or behavioral variability

Correlated variability is thought to arise through common input to a population of neurons. However, other factors may also affect the degree of measured r_SC._ Neuron pairs with higher firing rates tend to exhibit larger values of r_SC_, as do pairs that are physically closer^6,30,31^. To test whether these factors might account for our findings, we performed a mean-matching procedure^32,33^ to control for firing rate and distance between pairs (Methods). We found that even when these two factors were matched across neuronal classes, significant variation of r_SC_ across classes persisted (Supplementary Fig. 2a). We further tested whether the variation in r_SC_ across classes could be accounted for by variation in relationship between signal correlations (r_Signal_) and r_SC_^6,30^, and found no systematic variation corresponding to our measured r_SC_ across classes (Supplementary Fig. 2b).

Correlated variability can, in principle, be caused by trial-to-trial variation in behavior or motor plan, or in any factor that causes common fluctuations in neuronal activity. Our data from the primate SC are not immune to these concerns because SC neurons play a central role in the control of eye and head movements. Nevertheless, there are several reasons why our results cannot be explained away as an artifact of motor variability. First, the differences between classes of SC neurons were present well before any movement occurred. Second, classes of neurons with similar motor-related activity (i.e., the Visual-Movement and Movement classes) exhibited very different levels of r_SC_. In fact, Movement class neurons exhibited the lowest levels of r_SC_, even during the time of saccadic eye movement. Third, correlations between neuronal responses and saccadic reaction times could not explain our findings, as these were not significantly different across classes (*P* > 0.05, Kruskal–Wallis). And lastly, repeating our analyses of r_SC_ on subsets of trials—either on trials with the smallest saccadic accuracy or the highest—did not lead to significantly different results (*P* > 0.05, ANOVA). Thus, the differences in r_SC_ strength across neuronal classes were not explained by differences in firing rate, distance, r_Signal_ to r_SC_ relationship, or trial-to-trial variations in behavior, and instead, point towards distinct sources of common input to the functional classes of SC neurons.

### The dependence of r_SC_ on functional class was replicated using alternative classification strategies

Our method for neuronal classification has been used extensively in the past and is largely considered “classic”^27–29^. We chose this method over others to link to the existing literature on SC neuron classes as well as to associate the classes with specific circuits, given how different classes map onto specific locations within primate SC, inputs, and outputs^34^. However, it is important to note that the distinction between classes is not clear cut, and that response properties of a neuron lie on a spectrum^25^. It is therefore possible to classify neurons in several different ways. In comparing different classification techniques to the “classic” approach (Supplementary Fig. 3a), we found that the dependence of r_SC_ on class shown in Fig. 2 could also be replicated when neurons were classified using other strategies: alternative classification techniques similarly revealed a dependence of r_SC_ on class (or cluster, depending on the technique), but only when the classification was based on task-related responses (Supplementary Fig. 3b–d) or neuronal properties such as waveform (Supplementary Fig. 3e). r_SC_ did not depend on class (or cluster) when clustering was performed on task-irrelevant baseline activity (Supplementary Fig. 3f). These results further show how distinct clusters of neurons in SC form microcircuits with differing degrees of r_SC_ within them, regardless to whether they neatly map onto classic approaches or are revealed by more modern techniques.

### r_SC_ within and between functional classes

**Fig. 3:**
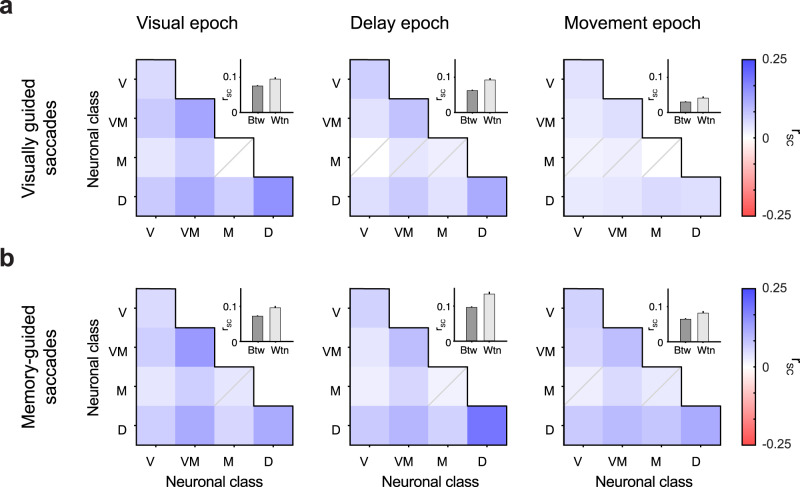
r_SC_ was larger within class than between classes. **a**, **b** Heatmaps of mean r_SC_ of neuron pairs between functional classes for visually guided (**a**) and memory-guided (**b**) saccades, one for each task epoch. Elements on the diagonal of the heatmap indicate within-class r_SC_ values (and consist of class-pairs: V-V, VM-VM, M-M, and D-D, where V, VM, M, and D stand for Visual, Visual-Movement, Movement, and Delay, respectively), off-diagonal elements indicate between-class r_SC_ (and consist of class-pairs: V-VM, V–M, V–D, VM-M, VM-D, and M-D). Elements for which the mean r_SC_ of neuronal pairs was not statistically significantly different from their trials-shuffled (null) distribution are indicated by a gray diagonal on the element (*P* > 0.05, Student’s *t* test, corrected). Insets show the mean r_SC_ across all within-class groups versus all between-class group for each epoch.

Having established that pairs of SC neurons exhibit differing degrees of r_SC_ within each functional class, we next assessed the degree of pairwise correlations between classes. We quantified the degree of r_SC_ between neurons belonging to different functional classes during the three epochs of the task (off-diagonal of the heatmap, Fig. 3). For reference, we also include the within-class correlation values (same data shown previously in Fig. 2e, f, appear on the diagonal of the heatmap).

Neurons exhibited positive r_SC_ values both within and between classes, primarily during the visual and delay epochs of the task, but the between-class values were smaller. During the visual epoch of visually guided saccades (Fig. 3a, left), high r_SC_ was observed for neurons within the Visual-Movement class, within the Delay class, and between them. Movement class neurons formed the weakest correlations across classes. A similar pattern was observed for r_SC_ during the delay epoch (Fig. 3a, middle). In contrast, correlated variability during the movement epoch was substantially reduced, both within and between classes (Fig. 3a, right). Overall, average within-class r_SC_ were higher than between-class (*P* < 0.001 in both epochs, Student’s *t* test). The degree of correlated variability for memory-guided saccades (Fig. 3b) followed a similar pattern to that observed for visually guided saccades, but again with a conspicuously higher r_SC_ for neurons within the Delay class during the delay epoch.

In addition to measuring r_SC_ between classes within epochs, we measured r_SC_ between epochs. This analysis sought to evaluate whether a transfer of information took place over time, but all between-epoch correlations were small and observed for only a limited number of class combinations (Supplementary Fig. 4).

Overall, the correlated variability exhibited by SC neurons was predominantly between pairs of neurons within the same functional class and during a given epoch. Pairs from different classes also exhibited significant levels of r_SC_, albeit to a lesser extent.

### r_SC_ in Delay class neurons was modulated by task demands and target location

**Fig. 4:**
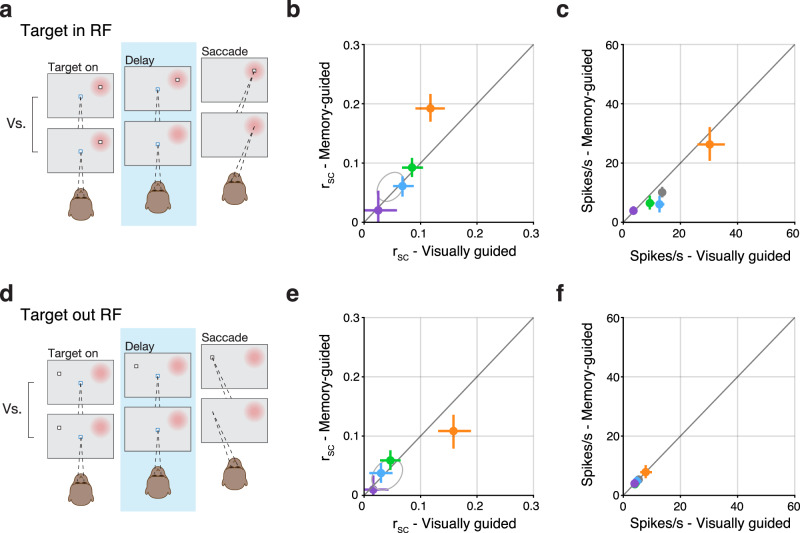
r_SC_ in Delay class neurons was modulated by saccade condition and target location, independent of firing rate. **a** Graphic indicting the comparison of visually guided versus memory-guided saccade trials during the delay epoch when the saccade target was presented within the RF of the recorded neurons (RF indicated by the red patch). **b** r_SC_ values in the memory-guided condition plotted against values in the visually guided condition. Error bars indicate bootstrapped 95% confidence intervals on the mean of r_SC_. *n* pairs are similar to those reported in Fig. 2. Gray ellipse reflects bootstrapped 95% confidence intervals on the mean of r_SC_ values for pairs drawn from a class-blind “null” distribution. **c** Mean firing rate (spikes/s) values in the memory-guided saccade condition plotted against mean firing rate in the visually guided condition. Error bars indicate bootstrapped 95% confidence intervals. **d**–**f** Same format as (**a**–**c**), but for when the saccade target was presented *outside* the RF of the recorded neurons.

The strong dependence of r_SC_ on the functional class during the delay epoch was larger for saccades that relied on working memory compared to those that did not. The qualitative difference between visually and memory-guided saccades motivated us to compare these data directly (Fig. 4). Juxtaposing the saccade conditions revealed that only Delay class neuron pairs displayed levels of r_SC_ that differed significantly between visually guided and memory-guided saccades (Fig. 4a, b, *P* < 0.001, two-way ANOVA). The relative increase in r_SC_ during memory-guided saccades cannot be due to a difference in firing rate because these were not significantly different between the two saccade conditions, and if anything, were lower for memory-guided saccades (Fig. 4c). Thus, Delay class neuron pairs stood out not only because r_SC_ in this class was uniquely modulated by saccade task demands, but also by virtue of exhibiting the largest level of r_SC_ overall.

The larger r_SC_ for Delay class neurons held true even when target location was *outside* their RFs (Fig. 4d, e). We found that for “out-RF” trials, during which the saccadic target was presented in the opposite hemifield, SC neurons exhibited a similar pattern to that observed for in-RF trials (Fig. 4b) in that Delay class neurons exhibited the highest level of r_SC_ and were uniquely modulated by saccadic condition (*P* < 0.001, two-way ANOVA). A key difference between the out-RF and in-RF data, however, is that the sign of the condition-related modulation was inverted: for in-RF trials, Delay class r_SC_ was larger for memory-guided saccades compared to visually guided (Fig. 4b), but for out-RF trials, was smaller (Fig. 4d). Here too, the difference in r_SC_ cannot be attributed to a difference in firing rate because spike counts were not significantly different between visually and memory-guided saccades (Fig. 4e).

Thus, the level of r_SC_ in Delay class neurons depended both on saccade condition and on target location, consistent with a spatially selective modulatory input related to the difference in task demands between memory versus visually guided saccades.

### No significant r_SC_ detected across hemispheres

**Fig. 5:**
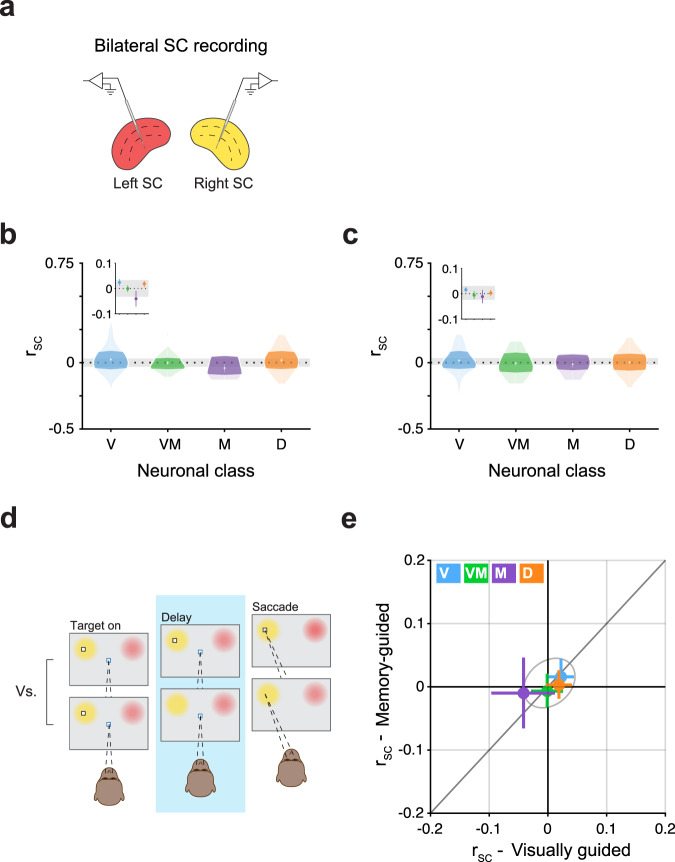
No r_SC_ detected across hemispheres. **a** Schematic of bilateral recordings. One multichannel probe was positioned in each SC, simultaneously recording bilateral pairs of SC neurons. **b**, **c** Violin plots show the r_SC_ values for bilateral pairs in each neuronal class during the delay epoch of visually guided (**b**) and memory-guided (**c**) saccades. Darker regions indicate the interquartile range. Gray horizontal bar reflects 95% confidence intervals on the mean of r_SC_ values for pairs drawn from a class-blind “null” distribution, bootstrapped. Insets zoom-in on functional classes’ means with 1 SEM error bars, bootstrapped. *n* bilateral pairs = 84, 36, 19, and 63 for the Visual, Visual-Movement, Movement, and Delay class, respectively. **d** Graphic illustrating the comparison of visually guided versus memory-guided saccade trials during the delay epoch when the saccade target was presented in either the right or left set of neuronal RFs, corresponding to either the left or right SC (the graphic shows the target on the left, but trials from both locations were used). **e** r_SC_ values in the memory-guided condition plotted against values in the visually guided condition. Error bars indicate bootstrapped 95% confidence intervals on the mean of r_SC_. Gray ellipse indicates bootstrapped 95% confidence intervals on the mean of r_SC_ values for bilateral pairs drawn from a class-blind “null” distribution.

The dependence of r_SC_ on target location motivated us to test whether our findings extended to SC neurons across hemispheres. We hypothesized that the relative inversion of Delay class r_SC_ during visual versus memory-guided saccades as a function of target location might indicate inter-hemispheric competition amongst Delay class neurons. On a subset of sessions (*n* = 6 in one monkey), we recorded from the left and right SC simultaneously (Fig. 5a). We used these bilateral neuron pairs to test whether certain functional classes exhibited correlated (or anti-correlated) activity between the two SCs. For example, the presence of inter-hemispheric competition might be expected to produce negative r_SC_ values.

We found no significant cross-hemisphere r_SC_ for either functional class, regardless of saccade condition (Fig. 5b and c). For each class, no significant difference between the delay epoch r_SC_ and the trial-shuffled (null) distribution (*P* > 0.05, Student’s *t* test) was detected, in either visually or memory-guided saccade trials. Likewise, no significant differences were found between classes in either saccade condition (all *P* > 0.05, ANOVA). Thus, the correlated variability across bilateral pairs in all classes and saccade conditions was statistically indistinguishable from zero and bounded by values obtained in a power analysis (see Methods), indicating that common input onto SC neurons was neither correlated nor anti-correlated, and largely independent across the two hemispheres.

As opposed to r_SC_ within a single SC, where significant differences were observed between classes that depended on saccade condition and target location (Fig. 4), no differences between saccade conditions were observed for cross-hemisphere r_SC_, in any functional class (Fig. 5e, all *P* > 0.05, Student’s *t* test). Thus, the modulatory effect of saccade condition on Delay class r_SC_ (Fig. 4) likely operates on the left and right SC independently, with little to no competition across hemispheres (Fig.5).

### Delay class neurons exhibited the longest temporal autocorrelations

Finally, we assessed whether the intrinsic timescale of neuronal activity^35^ might also differ across the classes of SC neurons, based on the premise that different functions might involve different temporal dynamics. We adopted an approach used previously^35^ and applied it to the different classes of SC. Unlike our previous analyses that focused on either visual, delay or movement epochs of the saccade tasks, this analysis was restricted to a 500 ms epoch before target onset and during fixation, termed the “foreperiod”. Temporal autocorrelation during this foreperiod can be used to gain insight into the intrinsic timescale of a circuit, independent of task-related factors such as saccade condition or target location.

**Fig. 6:**
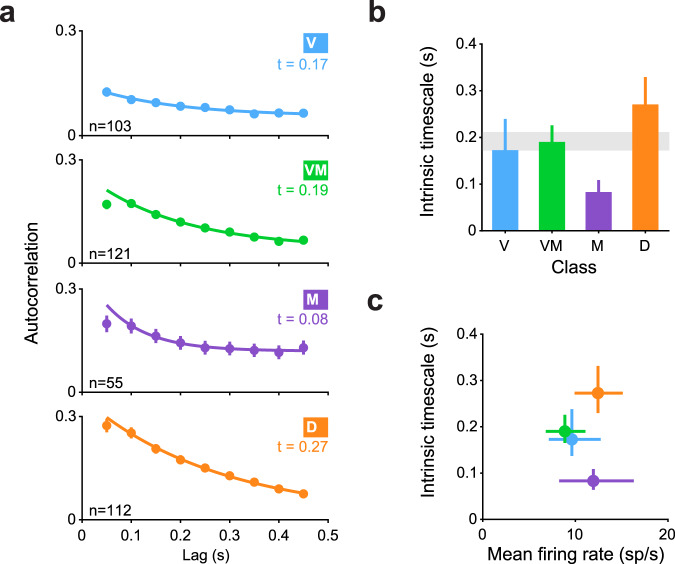
Temporal autocorrelations were the longest in Delay class neurons. **a** Spike-count autocorrelations for each functional class of SC neurons. Autocorrelations were computed in 10 non-overlapping, successive 50 ms bins during the foreperiod (during fixation, prior to target onset). Error bars indicate 1 SEM, bootstrapped. Sample n are indicated on the panels. Solid line represents the fit of an exponential decay with an offset. Timescale of decay (“*t*”) is noted on the panel of each class. **b** Intrinsic timescale of decay for each class. Error bars indicate bootstrapped 68.2% confidence intervals (1 SEM) on the timescale parameter estimate. Gray bar indicates bootstrapped 68.2% confidence intervals on the timescale parameter estimate computed over all SC neurons irrespective of class. **c** Timescale of decay for each class as a function of mean firing rate. Vertical error bars are identical to those in (**c**), horizontal error bars indicate 1 SEM, bootstrapped.

On average, neurons from each class exhibited a general decay pattern characteristic of a temporal autocorrelation function (Fig. 6a). A similar pattern was observed for individual neurons and over all time bins (Supplementary Fig. 5a, b, respectively). We fit a decaying exponential to the average data to quantify the intrinsic timescale. Timescales differed significantly across classes (Fig. 6b, *P* < 0.001, Kruskal–Wallis), with Movement class neurons exhibiting the shortest intrinsic timescale and Delay class neurons exhibiting the longest, in line with the functional specializations of these classes of neurons. This difference in timescales across classes could not be explained as a byproduct of firing rate, as these were similar across classes during the foreperiod (Fig. 6c). Taken together, results from this analysis of single units complement those obtained by the analysis of pairs, whereby both intrinsic timescales and r_SC_ were different across the functional classes of SC, and most pronounced in neurons belonging to the Delay class.

## Discussion

Correlated variability (spike-count correlations, r_SC_) is widely reported in the literature and plays an important role in shaping information transfer in the brain^6,8–10^. In this paper, we demonstrated that r_SC_ depends on functional class. First, r_SC_ within a functionally identified subpopulation of neurons was strikingly different from the r_SC_ for the overall population of neurons (Fig. 2). Second, the magnitudes of r_SC_ varied across different epochs of the task for neurons both within and between classes (Fig. 3). Third, Delay class neurons stood out as particularly correlated during the delay epoch of the saccade task, the magnitude of which was modulated by whether the saccade relied on working memory or not (i.e., memory or visually guided), and by whether it was directed into or out of the neuronal RFs (Fig. 4). Fourth, despite the dependence of Delay class r_SC_ on saccade direction, no significant r_SC_ (neither positive nor negative) was detected for pairs of Delay class neurons (or for any other class) across hemispheres (Fig. 5). Fifth, this dependence of r_SC_ on functional class cannot be trivially explained as a byproduct of motor activity, since the magnitude of r_SC_ was lowest in the Movement class of neurons. Lastly, Delay class neurons exhibited the largest temporal autocorrelations across all classes (Fig. 6).

### Distinct levels of r_SC_ in the SC

Our main finding, that different functional classes in SC exhibit distinct levels of r_SC_ (Fig. 2), highlights the importance of taking functional class into account when measuring and interpreting r_SC._ The current study investigated primate SC, but microcircuits reflecting distinct functions, processing stages, or associations with feedforward or feedback projections exist in most areas of the brain^36–39^. Thus, while existing measurements of r_SC_ in many brain regions provide important information on the level of coordinated activity over all neuron pairs^6^, it is important to consider the possibility that this overall level may obfuscate the actual r_SC_ of constituent subpopulations or microcircuits within the region. This is especially important in early sensory areas where the measured r_SC_ is taken to inform (and constrain) downstream computations that support behavior, and even more consequential if it is the output neurons of a circuit that exhibit r_SC_ levels different from the average of the population.

Why would different classes of SC neurons exhibit distinct levels of r_SC_? Traditionally, the source of r_SC_ was thought to arise from shared noise on the sensory inputs, but recent evidence from the sensory cortex has shown that feedback projections play a substantial role in shaping the structure of r_SC_^11,18,40^. Indeed, cortical laminae associated with different circuit positions and functions possess distinct degrees of r_SC_^41,42^. Thus, different levels of r_SC_ in a neuronal population correspond to distinct inputs that can be either correlated or decorrelated^43^. Our data are consistent with this idea given that different classes of SC neurons reflect distinct functions, operate on different timescales (Fig. 6), and are associated with different inputs (reviewed in refs. ^23,26^).

For example, neurons in the superficial layers of SC (predominantly classified as Visual) receive a preponderance of feedforward inputs from early processing stages such as retinal ganglion cells, thalamus, and striate cortex, whereas neurons in the intermediate layers (predominately classified as Visual-Movement and Delay) receive inputs from numerous cortical areas, likely involving cortico-subcortical loops and task-related feedback projections. Such a functional architecture is consistent with our finding that changing the saccadic condition from visually guided to memory-guided, which relies on working memory and is thought to engage feedback or recurrent activity^44–47^ resulted in different levels of r_SC_ in Delay neuron pairs, but not others (Fig. 4). Unlike the high r_SC_ observed in Delay class neurons, Movement neurons exhibited a relatively low level of r_SC_, across all epochs of the task. Lower r_SC_, often associated with increased information, may be related to reducing movement error in this set of neurons. Task designs in which monkeys are rewarded for higher movement accuracy might be useful in testing this hypothesis. Identifying different levels of r_SC_ across classes does not reveal their significance. But the differences observed are informative and potentially consequential, especially since the magnitude of differences found here were at least as large as the changes in r_SC_ implicated in functions such as perceptual learning and selective attention^22^.

Distinct functional classes have also been identified in FEF and LIP neurons^48,49^, but a systematic measurement of r_SC_ in each has not been performed. In one related study, distinct levels of r_SC_ were reported in neuronal subpopulations of FEF binned along a visuomotor spectrum^50^, consistent with the results reported here. Two important differences, however, are that the study did not include Delay class neurons as a separate functional class in its analysis, and it used only memory-guided saccades, thereby precluding an evaluation of whether r_SC_ in some neuronal classes are differentially affected by task manipulations. It is possible that our finding of higher r_SC_ levels in SC Delay class neurons would extend to the analogous classes of neurons in FEF and LIP as well. It is further possible that the higher r_SC_ exhibited within vs. between class (Fig. 3) extends to pairs of neurons across areas, such that Delay class neurons in oculomotor areas SC, FEF, and LIP, for example, correlate with one another to a larger extent. Lastly, the excitatory or inhibitory nature of neurons in these areas may also play a role in shaping r_SC_ as well as its dependence on task demands, as was observed in area V4^51^.

### The effect of task demands on r_SC_ in Delay class neurons

Experimental manipulations aimed at influencing cognitive states have significant effects on r_SC_ in many brain regions^6,52^. In the current study, manipulating whether the subject was to perform a visually or memory-guided saccade also influenced r_SC_, but only in Delay class neurons (Fig. 4b). Why did Delay class neurons exhibit higher r_SC_ during the delay epoch of memory-guided saccades compared to visually guided? One interpretation is that reliance on working memory in the memory-guided saccade task is associated with higher r_SC_. A second interpretation is that the visual target in the visually guided condition drives a decorrelation in these neurons. Stimuli presented in neurons’ receptive fields have been associated with decorrelation^30,32,53^, but it is unclear why this phenomenon should occur in Delay class neurons specifically, and not in neurons from other classes that process visual information. Given the class-specific nature of this effect, we believe the change in r_SC_ is likely linked to a function specific to Delay class neurons, and not to a general one like visual processing. Thus, it seems more likely that the relative increase of r_SC_ in Delay class neurons is due to correlated input related to the maintenance of target position in working memory.

What might be the source of such correlated input to Delay class neurons during working memory periods? If correlations were present in the sensory input (that is, due to “noise correlations” as originally defined), then higher r_SC_ in Delay class neurons would indicate that the accuracy of the information they provide about target location would be limited by this shared noise. An alternate view, which we favor, is that the r_SC_ is introduced specifically onto these neurons by other circuits in the brain to meet a behavioral goal, such as maintaining target position in working memory, consistent with recent evidence that r_SC_ are task-dependent^11^. Prefrontal and parietal neurons are candidate sources of such correlated input, as these cortices are functionally connected to SC^54–58^, have long been associated with working memory^45,46,59^, and exhibit the same type of persistent activity displayed by SC Delay class neurons: activity during the delay period between vision and action, in the absence of a stimulus in the neuronal RF^27,60–62^. Such an increase in r_SC_ is also consistent with a “*transmitter-receiver*” architecture^63^, in which case increased r_SC_ is indicative of improved communication. Thus, corticotectal inputs from prefrontal and parietal areas could underlie our observation of high r_SC_ in Delay class neurons that is modulated by the reliance on working memory given the saccade condition.

The direction of r_SC_ modulation by saccade condition depended on whether the saccadic target was in the RF of the neurons under study. When saccades were directed into the RF, r_SC_ was high for memory-guided saccades and low for visually guided (Fig. 4b) but when saccades were directed to the opposite hemifield, the reverse was observed (Fig. 4e). If input from upstream regions such as prefrontal cortex is the source of increased r_SC_ during memory-guided saccades into the RF, then a similar input must reach the SC on the other side of the brain during out-RF saccades. Such an asymmetry in modulatory input is consistent with inter-hemispheric competition and may produce negative r_SC_ values across the two colliculi, but this was not observed in either class (Fig. 5). Thus, the modulatory input onto Delay class neurons appears largely independent across the left and right SC, consistent with the idea that visual working memory operates independently across hemispheres^64,65^. If the source of such modulatory input is cortical, this indicates that two independent processes are operating in cortex to hold target position in working memory, even though the target appeared in only one hemifield.

### Difference in r_SC_ across classes could not be explained as byproducts of other factors

Measurements of r_SC_ are sensitive to a number of confounding factors such as firing rate and physical distance^6^. We performed several control analyses to rule out such confounds and found that our main results were unchanged (Supplementary Fig. 2 and Fig. 4c, f). Another potential confound in our measurements is trial-to-trial variability in motor responses—for example, in small-amplitude fixational eye movements termed microsaccades. Moving the eyes can synchronously modulate the spiking of simultaneously recorded neurons, providing a possible source of pairwise correlations^66^. However, movement of the eyes would be expected to primarily affect neurons with visual or movement properties such as neurons from the Visual, Movement, and Visual-Movement classes, and yet these had lower r_SC_ values than neurons from the Delay class. Moreover, we found that Movement class neurons, which would be expected to be most influenced by trial-to-trial variability in movements, had the lowest r_SC_ of all classes. In other words, trial-to-trial variations in saccadic reaction time and endpoint did not manifest in increased r_SC_ during the movement epoch, for Movement class neurons or any others.

Increased r_SC_ in neurons might also be due to RF size. Trial-to-trial variations would have larger effects on neuronal firing rates if RFs are small, and smaller effects on firing rates if RFs are large. This would translate into higher values of r_SC_ for small RFs and lower values of r_SC_ for large RFs (and negative r_SC_ values if the RFs are non-overlapping, but this scenario does not apply to our work since we only included neurons with overlapping RFs). If differences in RF size explained our results, one would expect Delay class neurons (which exhibit a relatively high r_SC_) to have the smallest RF sizes, and Visual class neurons (which exhibit a relatively low r_SC_) to have the largest RFs. Contrary to this prediction, previous studies have reported the opposite: that neurons in the intermediate and deep layers of the SC (where a preponderance of Delay class neurons are found) have larger RFs than those in the superficial layers (where a preponderance of Visual class neurons are found)^67,68^. Thus, differences in RF sizes across classes cannot explain the different levels of r_SC_ we measured across functional classes.

We also considered whether the higher r_SC_ in Delay neurons during memory-guided saccade trials (Fig. 4) was due to a larger proportion of microsaccades or other behavior not specifically controlled for during these trials compared to visually guided. But again, this explanation would seem to imply increases in r_SC_ in all classes during memory-guided saccade trials, not just Delay class neurons. And would imply similar effects on r_SC_ for both in-RF and out-RF trials, and in both colliculi, in contradiction to our findings (Figs. 4 and 5). The same argument can be used to rule out global factors related to arousal, as mechanisms related to arousal would be expected to influence both in-RF and out-RF trial types, as well as both colliculi. Thus, while the occurrence of microsaccades and non-specific arousal signals may affect overall levels of r_SC_, it is unlikely that these could account for the observed differences across classes and tasks.

### Summary

As recording techniques advance and larger pools of neurons are recorded simultaneously, considering r_SC_ as a metric of functional connectivity has become standard practice. Gaining better insight into what constitutes a population of neurons or whether a population is composed of several subpopulations is important for accurately measuring r_SC_ in a brain area and informing population coding. Here we found that neurons identified by functional class exhibited very different levels of r_SC_ from the population average as well as from one another, presumably because each subpopulation of neurons occupies a distinct circuit niche within the SC network. Our findings highlight the importance of taking neuronal class into account when measuring correlated variability or interpreting its significance in primate SC, or elsewhere in the brain.

## Methods

### Animals

Two adult male rhesus monkeys (*Macaca mulatta*) weighing 9–12 kg were used in the study. All experimental protocols were approved by the National Eye Institute Animal Care and Use Committee and all procedures were performed in accordance with the United States Public Health Service policy on the humane care and use of laboratory animals. A plastic headpost and recording chamber had been previously implanted granting electrophysiological access to the superior colliculus (SC).

### Experimental apparatus

Animals were seated and head-fixed in a primate chair (Crist instrument Inc) inside a darkened booth facing a 100 Hz VIEWPixx display (VPixx Technologies) that was controlled by a mid-2010 Mac Pro (Apple Inc) running MATLAB (The Mathworks) with the Psychophysics Toolbox extensions^69^. Eye position was recorded using an EyeLink 1000 infrared eye-tracking system (SR Research Ltd.) and monitored online for gaze-contingent progression through the task. Experiments were controlled using a modified version of PLDAPS^70^.

### Guided saccade task

Animals performed a saccade task that included both visually guided and memory-guided saccade trials (Fig. 1b). In either version of the task, a trial began with the appearance of a 0.25° wide white fixation square (48 cd/m^2^) on a gray background (28.5 cd/m^2^). Fixation had to be maintained within a 2° wide square window (invisible to the monkey). At 0.5–0.7 s following fixation acquisition, a 0.25° wide square-shaped white saccade target (48 cd/m^2^) appeared in one of four possible locations and either stayed on (visually guided condition) or was extinguished following 0.2 s (memory-guided condition). Monkeys were required to maintain fixation during target presentation up until the disappearance of the fixation square (the “go signal”, 1–2 s following target onset), whereupon a saccade was to be executed towards the target within 0.1–0.5 s, land within a 3° wide window around the target (invisible to the monkey) and maintained within the window for a duration of 0.5–0.7 s. In the memory-guided condition, the target was reillumined following entrance into the target window for a duration of 0.2 s. In the visually guided saccade task, 74.5% of trials were completed successfully. In the memory-guided saccade task, 73.5% of trials were completed successfully. Successful completion of a trial resulted in a juice reward while any deviation aborted the trial and trial identity (i.e., target location and condition) was reshuffled into the block of trials. The precise timings of events on every trial were determined by random draws from a uniform distribution within the prescribed ranges.

Each block consisted of 24 trials. On every trial, a target appeared in one of four locations. In the hemifield contralateral to a recorded SC, one target location was positioned to maximally overlap with the response fields (RF) of neurons, and another at a 90° angle away from the first either above or below the horizontal median at a similar radius, within the same hemifield. In sessions where only one SC was recorded from, targets on the hemifield ipsilateral to the recorded SC (where no RFs were present) were positioned diametrically opposite to those in the contralateral. Trials were distributed at a proportion of 2:1 for each pair of targets within a hemifield, where targets placed in neuronal RFs (or diametrically opposite targets) were used more frequently. Targets in neuronal RFs ranged from 4° to 23° in eccentricity (median of 10°), and spanned a variety of angles. The trial condition was distributed equally (i.e., 50% visually guided and 50% memory-guided) for each target location. Overall, a typical session consisted of 20 blocks and netted 516 ± 126 trials (mean ± std).

### Electrophysiological recordings

Electrophysiological signals and eye position were acquired by an Omniplex-D system (Plexon Inc.). Neuronal activity in SC was recorded using 32-channel Plexon v-probes with 50 µ inter-channel spacing (Plexon Inc.) from either one of the SCs or both simultaneously, with one probe in each SC. Probes were advanced to their target depth in the intermediate and deep layers of the SC using a motorized microdrive (NAN Instruments). Target depth was guided by the previous mapping and verified by measuring neuronal responses online. Activity on each channel of the probe was thresholded (µ – 3σ) and used to map the spatial RF by having the animal perform visually guided saccades to targets presented at locations drawn randomly from an XY grid (5° spacing from −25° to 25° on the X-axis, and −15° to 15° on the Y) as well as locations set manually by the experimenter to more precisely measure the spatial extent of the RF. A saccade target was then positioned in the center of the RFs and used to collect several memory-guided saccades (typically ~20) sufficient to ascertain the probe’s location within the SC: visual responses were associated with superficial SC layers; saccade-related activity with deep. Probe position was then adjusted to maximize neuronal yield from intermediate and deep SC layers, and left to settle (~1 h) to allow the tissue to stabilize before beginning the experimental session.

### Electrophysiological analysis

Only data recorded during successfully completed trials were used for analysis. Continuous spike-channel data collected during the experimental session were sorted offline with Kilosort2^71^ using in house tools (https://github.com/ElKatz/kilo2Tools), and manually curated by a human expert using Phy2 to ensure that all sorted units have plausible inter-spike interval distributions and waveform shapes consistent with action potentials. Neurons with low (<1.8) signal-to-noise ratio^72^ were excluded. Increasing the signal-noise threshold to 2 or 2.5 did not change our main results, but reduced statistical power substantially. Offline, we determined the “in-RF” target for each neuron as the target that elicited the largest visual (+50 to +200 ms relative to target onset) or movement (25 ms before saccade onset to saccade end) responses for each neuron. If the visual or movement-related responses were not significantly larger than baseline (−75 to +25 relative to target onset) using an ANOVA (alpha = 0.05, corrected), the neuron was not associated with an in-RF target. Only neurons associated with an in-RF target were used for subsequent analysis. We further excluded low-firing neurons (<0.1 spikes/s averaged over all targets and time) as r_SC_ estimates for low-firing neurons tends to be poor^6^. Increasing this exclusion criterion to 1 or 5 spikes/s did not change the main results. Overall, 908 neurons were recorded (366 from Monkey #1, 542 from Monkey #2) and 209 of these did not meet our inclusion criteria, netting 699 neurons for the analysis reported here (314 from Monkey #1, 385 from Monkey #2). Results did not differ across monkeys and were therefore combined to increase statistical power.

For the visualization of mean firing rated over time relative to key events in the task (Fig. 1e), normalized spike counts were binned into non-overlapping 20 ms bins. Each neuron’s data was *z*-scored normalized by subtracting the mean and dividing its binned spike counts by the standard deviation of that neuron’s counts across trials and conditions.

### Neuronal classification

Each neuron was assigned a functional class following established criteria^27–29^. Briefly, spike counts during four epochs in the memory-guided saccades to the in-RF target were used to determine neuronal class: baseline epoch (−75 to +25 ms relative to target onset); visual epoch (+50 to +200 ms relative to target onset); delay epoch (−150 to +50 relative to fixation offset); movement epoch (−25 ms from saccade onset to saccade offset). Saccade onsets and offsets were calculated offline using a velocity threshold (30°/s) and verified by inspection. A one-way Kruskal–Wallis nonparametric ANOVA was used on spike counts in the four epochs to determine whether a neuron possessed visual-, delay-, or movement-related responses. Such an analysis netted seven possible classes: visual (*v*), visual-delay (*vd*), visual-movement (*vm*), visual-delay-movement (*vdm*), delay (*d*), delay-movement (*dm*), and movement (*m*). This classification was used to construct four operational classes that correspond to past classifications of SC neurons: a “Visual” class, consisting of *v* neurons (28% of our population); a “Visual-Movement” class, consisting of *vm* neurons (27%); a “Movement” class, consisting of *m* neurons (15%); and a “Delay” class, consisting of all neurons that exhibited delay-related activity: *vd* (6%); *vdm* (9%)*; d* (1%); and *dm* (2%). The Delay class is similar to “prelude” (or “build up”) neurons^27–29,60,73^ as it includes similar groups of neurons (the *vdm* and *dm*), but is distinct in that the Delay class used here also includes *vd* and *d* neurons. Whether or not our Delay class included all delay-exhibiting neurons (*vd*, *vdm*, *d* and *dm*) or only a subset corresponding to the classically defined “prelude neurons” (*vdm* and *dm*) did not change any of our main results (see “Delay class subset” in Supplementary Fig. 2a). This indicates that the number of epochs to which the neuron was responsive to does not influence the degree of r_SC_ for that class. Likewise, the degree of r_SC_ in our dataset did not depend on firing rate (Supplementary Fig. 2a and Fig. 4c, e).

Overall, our neuronal dataset includes 699 neurons, of which 198 were classified as Visual, 187 as Visual-movement, 103 as Movement, and 135 as Delay (Fig. 1d). In total, 76 neurons exhibited no distinct activity in any of the task epochs and were not used for further analysis.

### Spike-count correlation (r_SC_) measurements

Spike-count correlations between pairs of simultaneously recorded neurons (r_SC_) were defined as the Pearson’s correlation coefficient of spike counts during repeated instances of the same task conditions, calculated as:

1 $${r}_{SC}=\frac{E\left[{N}_{1}{N}_{2}\right]-E{N}_{1}{{EN}}_{2}}{{\sigma }_{{N}_{1}}{\sigma }_{{N}_{2}}}$$

where ${N}_{1}$ and ${N}_{2}$ are the spike counts for neurons 1 and 2 (respectively), $E$ is the expected value of the counts, and $\sigma$ is the standard deviation. We followed standard practice to avoid contamination in estimations of r_SC_ values by removing trials with response outliers (>3 SDs difference from the mean)^1,30^. All statistical evaluations of r_SC_ values within or between classes were performed following the Fisher r-to-z transformation to approximate normality and stabilize variance in the data^30,74^. Untransformed r_SC_ values were only presented when visualizing individual pairs (supplementary Figs. 1 and 2b).

Only neurons with overlapping RFs were included in our analysis of pairwise correlations, netting 4626 pairs overall. For pairs of neurons within the same class, we obtained 477, 470, 104, and 269 pairs for the Visual, Visual-movement, Movement, and Delay classes, respectively. r_SC_ values were computed within time bins of 150 ms: 0–150 ms after target onset (the visual epoch), 650–800 ms after target onset (the delay epoch), and 75 ms before the saccade to 75 ms after (the movement epoch) (see shaded regions in Fig. 2a). Whether our delay epoch window was at 650–800 ms after target onset, earlier (500–650 ms), or later (800–950 ms), did not change the results. For measurements of r_SC_ over time (time courses in Fig. 2a–d), we used the same bin size (150 ms) in a sliding window with 50 ms increments.

### Spike-count autocorrelation measurements

Measurements of spike-count autocorrelation (Fig. 6 and supplementary Fig. 5) were performed following a procedure described previously^35^. Analysis was performed on the foreperiod, a 500 ms epoch during the fixation period that preceded target onset. In this epoch, animals were in a controlled state of preparedness with no variation in sensorimotor features of the task across trials. The foreperiod was divided into 10 separate, successive 50 ms bins and the Pearson’s correlation coefficient was computed between each $i$-th and $j$*-*th bin. For a single neuron or the average over a population, this procedure produces an autocorrelation matrix (Supplementary Fig. 5a). Decay of the temporal autocorrelation was computed by fitting a decaying exponential with an offset as a function of the time lag $k\Delta$ between bins ($k=\left|i-j\right|$):

2 $$\,R\left(k\triangle \right)=A\left[{{\exp }}\left(-\frac{k\Delta }{\tau }\right)+B\right]$$

where $\tau$ is the time constant reflecting the intrinsic timescale of decay, and $B$ is the offset. Some neurons exhibited a lower autocorrelation in the first time lag (50 ms) compared to subsequent lags, consistent with refractoriness. This has been noted previously^35^ and overcome by fitting the exponential decay to data starting at the largest reduction in autocorrelation between two consecutive time bins and onwards. Temporal autocorrelations in Fig. 6 were averaged over neurons and lags. Temporal autocorrelations averaged over neurons but not lags, and over lags but not neurons, are presented in Supplementary Fig. 5a,b, respectively.

### Pairwise signal correlation measurement

Measurements of signal correlations across pairs of simultaneously recorded neurons (Supplementary Fig. 2b) were performed on data obtained during the mapping of spatial RFs described above. In the task, visually guided saccades were executed towards many locations in space with variable proximity to the RF center of the neurons under study, thereby introducing variations in signal. Signal correlation was computed by calculating the Pearson correlation between responses of a pair of neurons during visually guided saccades (from target onset to saccade offset) to the range of targets presented.

### Statistical analyses

To determine whether different classes of SC neurons exhibited different levels of r_SC_ standard tests such as Student’s *t* and ANOVA were used (Bonferroni corrected for multiple comparisons). This choice was made because r_SC_ values used for statistical testing underwent a Fisher r-to-z transformation, which approximated normality and stabilized variance in the data^30,74^. Using nonparametric tests instead (e.g., Wilcoxon rank-sum or Kruskal–Wallis analysis of variance) did not change the results. Confidence intervals and SEMs were estimated using a standard bootstrap procedure with 10,000 random draws with replacement. To determine the statistical significance of a distribution of r_SC_ values the distribution was compared to a matched distribution in which trial identity was shuffled (Supplementary Fig. 1a). For class-blind “null” distributions (gray band or ellipse in Figs. 2g, h,  4c, e, and 5b, c), trial identity was not shuffled, but pairs were randomly sampled from the full dataset of 4626 neuronal pairs, irrespective of functional class.

To determine whether the reported differences in r_SC_ values across classes were artifacts of changes in firing rate or due to shorter distances between neurons, a mean-matching procedure^32,33^ was implemented (Supplementary Fig. 2a). Briefly, firing rates (or distances) of neuronal pairs were binned into 12 equally sized bins across all classes of neurons. From each bin, we randomly sampled (without replacement) a number of values that was determined by the bin with fewest data points, thereby creating sub-distributions with equal values across bins, matching the distribution of firing rates (or distances). This process was repeated 10,000 times to obtain the averages and SEMs presented in Supplementary Fig. 2a. Because Movement class neurons fired very sparsely during the delay epoch, their firing rate distribution did not overlap with the distributions of the other classes to a large enough extent, reducing the statistical power substantially. We therefore excluded the Movement class from the mean-matching procedure for firing rate (but not distance).

### Power analysis

In six sessions, we recorded from the SC bilaterally. Overall, 800 pairs of cross-hemisphere neurons were obtained, netting 84, 36, 19, and 63 bilateral pairs from the Visual, Visual-Movement, Movement and Delay functional classes, respectively. In our limited dataset, no correlations were detected for either functional class (Fig. 5). To determine the smallest absolute value of r_SC_ that our analysis method could detect with a confidence of 95%, we performed a bootstrapped power analysis. For each individual functional class, we simulated distributions of r_SC_ values with varying means (from −0.2 to 0.2, 0.005 increments) and a standard deviation determined by the standard deviation of the class-blind distribution of bilateral pairs (0.09). We randomly drew a number of samples equal to the number of pairs obtained in our recordings for that class and tested whether the mean r_SC_ was significantly different from zero (Student’s *t* test). We repeated this process 10,000 times to identify the mean r_SC_ value at which we could detect a statistical difference in 95% of cases. This approach determined the highest possible value of r_SC_ that could exist in our data and go undetected, with 95% confidence. For the Visual, Visual-Movement, Movement, and Delay classes, these values were 0.05, 0.08, 0.17, and 0.06 for visually guided saccades, and 0.04, 0.07, 0.16, and 0.05 for memory-guided saccades, respectively.

### Reporting summary

Further information on research design is available in the Nature Portfolio Reporting Summary linked to this article.

## Supplementary information

Katz_Peer Review File

Supplemental information

Reporting Summary

## Data Availability

Data for reproducing the main figures of the paper can be found in a DRYAD repository: https://datadryad.org/stash/dataset/doi:10.5061/dryad.12jm63z0r. For other data related to this study, please contact the corresponding author.

## Code availability

Code for generating main figures can be found in a DRYAD repository: https://datadryad.org/stash/dataset/doi:10.5061/dryad.12jm63z0r.
