# Supplementary material for: Correlated variability in primate superior colliculus depends on functional class
Source: Commun Biol. 2023 May 18;6:540. doi: 10.1038/s42003-023-04912-0 (PMC10195790; doi:10.1038/s42003-023-04912-0)
Supplement: Supplementary file 2 — Supplemental information [file 42003_2023_4912_MOESM2_ESM.pdf]

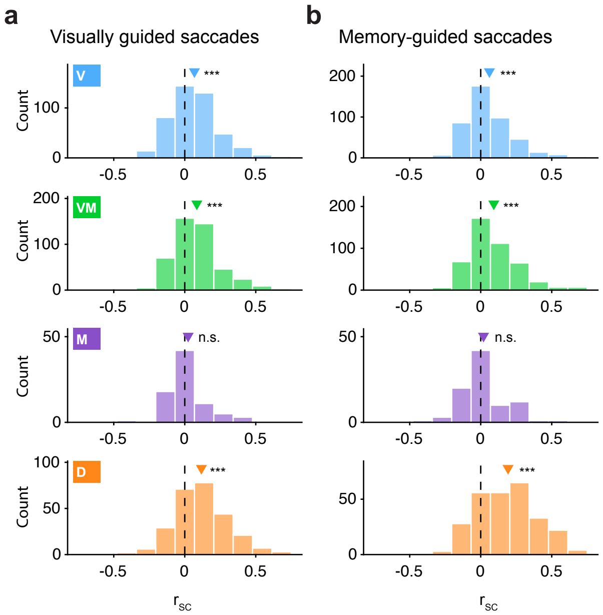

Supplementary figure 1: r_{SC} distributions over neuronal classes

Distributions of r_{SC} values within each neuronal class during the delay epoch of visually- and memory-guided saccades. These data were used to compute the mean r_{SC} and SEM displayed in Figure 2 g, h. **a** For visually guided saccades, distribution means (indicated by triangles on figure panels) ± s.d. for neuron pairs in the four classes were: 0.05 ± 0.17; 0.07 ± 0.18; 0.02 ± 0.14; and 0.12 ± 0.19. r_{SC} for all but the Movement class neurons differed significantly from a matched trial-shuffled (null) distribution (Student's t-test, asterisks on panel denote levels of statistical significance: *** $p < 0.001$; ** $p < 0.01$; * $p < 0.05$). **b** For memory-guided saccades, distribution means ± s.d. were: 0.06 ± 0.17; 0.09 ± 0.19; 0.02 ± 0.16; and 0.19 ± 0.23. r_{SC} for all but the Movement class neurons differed significantly from a matched trial-shuffled (null) distribution (same format as a).

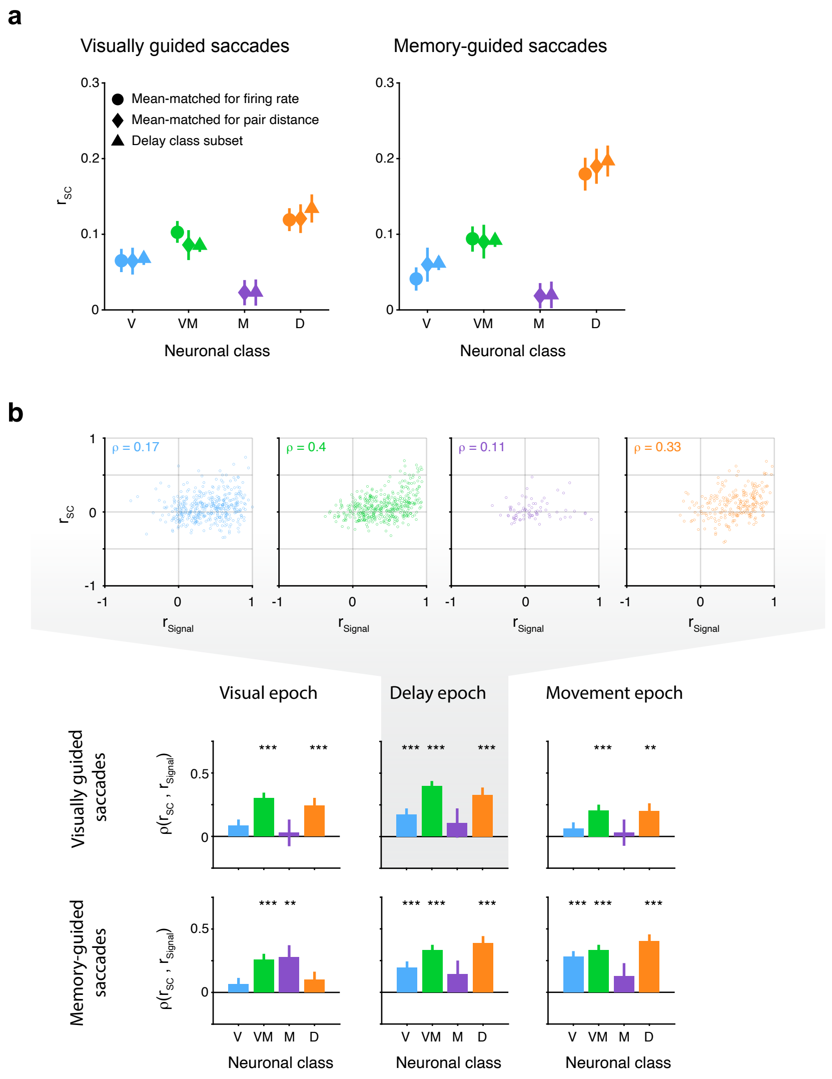

Supplementary figure 2: Dependence of r_{SC} on functional class was not due firing rate, distance or signal correlations

a We evaluated whether the variation in r_{SC} values across functional class presented in Figure 2g,h was influenced by firing rate, inter-pair distance, or details of functional classification method. We recomputed r_{SC} values during the delay epoch of either visually- (left) or memory-guided saccades (right) for three analysis variations: mean-matched firing rate; mean-matched distance; and Delay class subset. For mean-matched firing rate, the firing rates of each pair of neurons were matched across classes (excluding Movement class, see Methods) according to a standard mean-matching method (Methods). For mean-matched distance, the distance between each pair of neurons was matched across neuronal classes following the same mean-matching method. The location of each neuron was estimated as the channel on which its waveform amplitude was largest, and the distance of a pair was the distance between the two channels.

For the Delay class subset analysis, inclusion criteria for what constituted the Delay class differed to include only a subset of neurons within that class based on a different method for neuronal classification (See Methods for further details. Inclusion criteria for all other classes remained unchanged). In all variations of analysis method (firing rate, distance, or Delay class inclusion criteria), the significant difference across classes reported in Figure 2G, H was still observed, both for visually guided saccades ($p < 0.01$, $p < 0.05$, and $p < 0.001$, for the three analysis variations, respectively, ANOVA), and for memory-guided saccades ($p < 0.001$, $p < 0.05$, and $p < 0.001$, respectively, ANOVA).

b We evaluated whether the variation in r_{SC} values across functional class presented in Figure 2g,h was influenced by variations in the relationship between signal correlation (r_{Signal}) and r_{SC}. Top: the relationship between r_{Signal} and r_{SC} for each functional class. Pearson correlation coefficients ($\boldsymbol{\rho}$) between the two measurements are noted on the panels. Bottom: summary of the correlation coefficients for each class during the visual, delay and movement epochs (columns) and saccade conditions (rows). Asterisks above bars denote the statistical significance of the Pearson correlation: *** $p < 0.001$; ** $p < 0.01$; * $p < 0.05$.

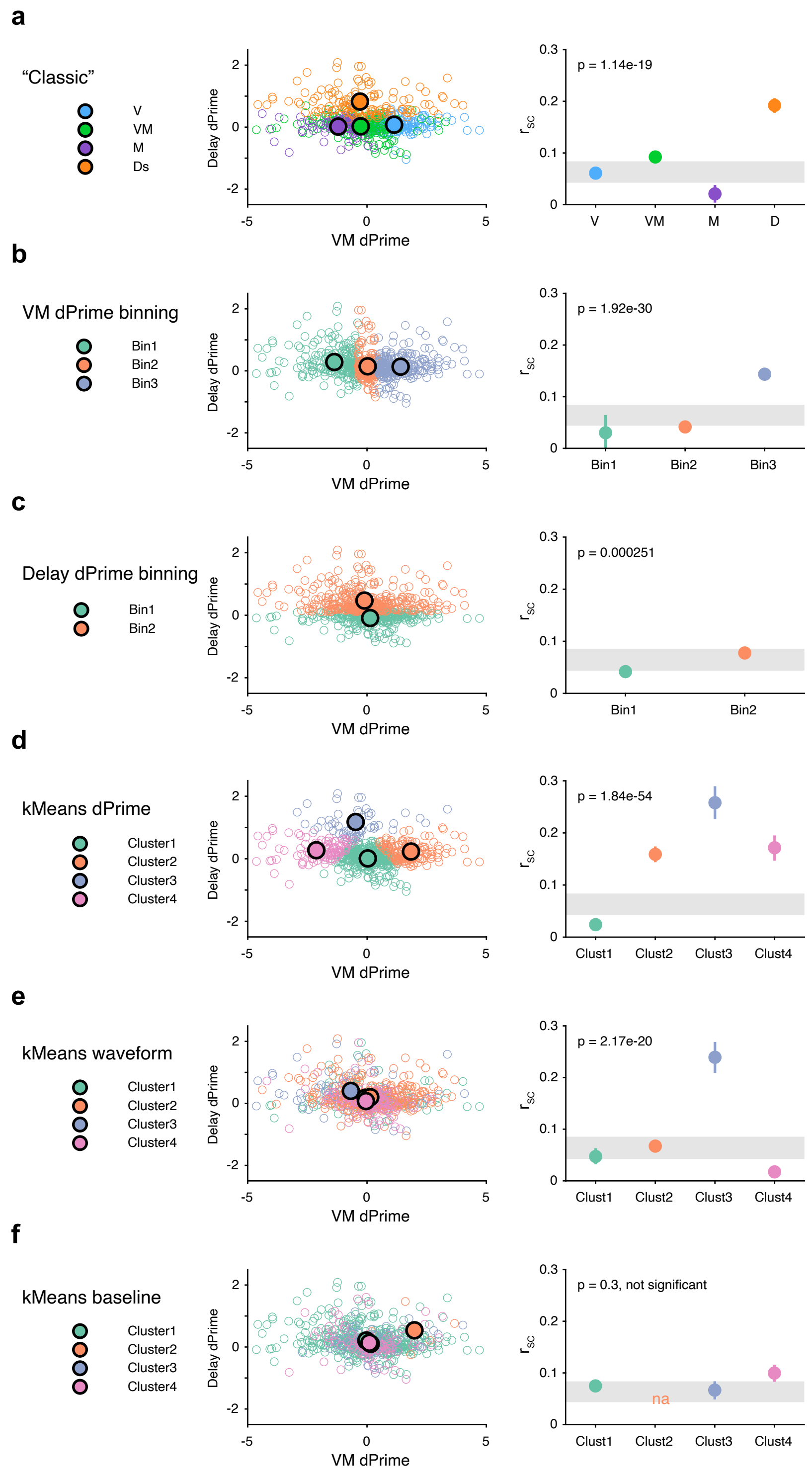

Supplementary figure 3: The dependence of rSC on functional class was replicated using alternative classification strategies

Each row in the figure corresponds to a different form of classification. To visualize how the classification maps on to known functional properties, we first constructed a 2D “dPrime space”, where visual-movement dPrime constitutes one axis (VM dPrime, following Khanna et al. 2009) and delay dPrime constitutes the other (Delay dPrime). **a “Classic”:** The first row shows results of our original classification (termed “classic classification”). Left: the classes used in this classification technique. Center: a visual representation of how this particular clustering approach maps onto dPrime space (large markers indicate means). Right: r_{SC} across classes during the delay period for memory guided saccades (for the “classic classification”, these data are similar to those presented in Main Figure 2H. Small variations expected due to bootstrapping. Error bars are 1 SEM, bootstrapped. Gray bar reflects 95% confidence intervals on the mean of r_{SC} values for pairs drawn from a class-blind random “null” distribution, bootstrapped). We focused on our main result— r_{SC} during the delay epoch of memory guided saccades—for the sake of clarity but note that results were similar for the other epochs (visual and movement) and task condition (visually guided saccades). **b VM dPrime binning**: Inspired by Khanna et al. 2009, we binned the Visual-Movement dPrime axis into discrete bins. We chose to use 3 bins because these may roughly map onto our original Visual, Visual-Movement and Movement classes, for comparison. Consistent with results in panel a, different bins of neurons exhibited different levels of r_{SC} (ANOVA p-value indicated on right panel), that were different than a “bin-blind” null set of pairs (gray bar in the panel). **c Delay dPrime binning**: Similar approach to panel b, but based on delay period activity. We chose to use 2 bins because these may roughly map onto our original classes: those with delay activity (i.e. Delay class neurons) versus those without (the rest). Consistent with results in panel a, different bins exhibited different levels of r_{SC} (Students t). **d kMeans dPrime:** we used unsupervised clustering (k-means, normalized) on the data in the dPrime space. We set k to 4 to compare results to our original clustering. The technique netted 4 clusters that loosely mapped onto the clusters obtained through classic classification (compare to panel a), where the cluster corresponding to Delay class neurons (“clust3”) exhibited the highest r_{SC} value. **e kMeans waveform:** we used unsupervised clustering (k-means, normalized) on the mean waveform shape of each neuron. Clusters did not map onto dPrime space in an interpretable way, but they significantly varied in r_{SC} value. **f kMeans baseline:** we used unsupervised clustering (k-means, normalized) on neurons’ activity during baseline activity (0 to 0.3 s following fixation acquisition), which is well before the target appears. In this task-irrelevant epoch, r_{SC} values did not significantly differ from one another, or from the “null” distribution of randomly selected pairs. “na” indicates insufficient data to plot the mean r_{SC}. Only clusters with >50 pairs are plotted.

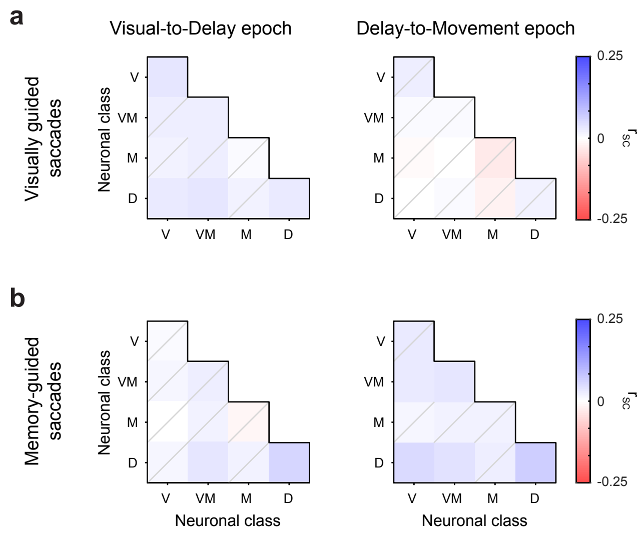

Supplementary Figure 4: r_{SC} across epochs were present in only a subset of neuronal classes

a-b Heatmaps indicating the mean r_{SC} of neuron pairs between functional classes for visually guided (a) and memory-guided (b) saccades, across epochs. Heatmaps on the left indicate the r_{SC} measured in neuron pairs between the visual and delay epochs, heatmaps on the right indicate the r_{SC} measured in pairs between the delay and movement epochs. Elements on the diagonal of the heatmap indicate within-class r_{SC} values, off diagonal elements indicate between-class r_{SC}. Elements for which the measured r_{SC} was not statistically significantly different from their trials-shuffled (null) distribution are indicated by a gray diagonal on the element ($p > 0.05$, Student's t test, Bonferroni corrected).

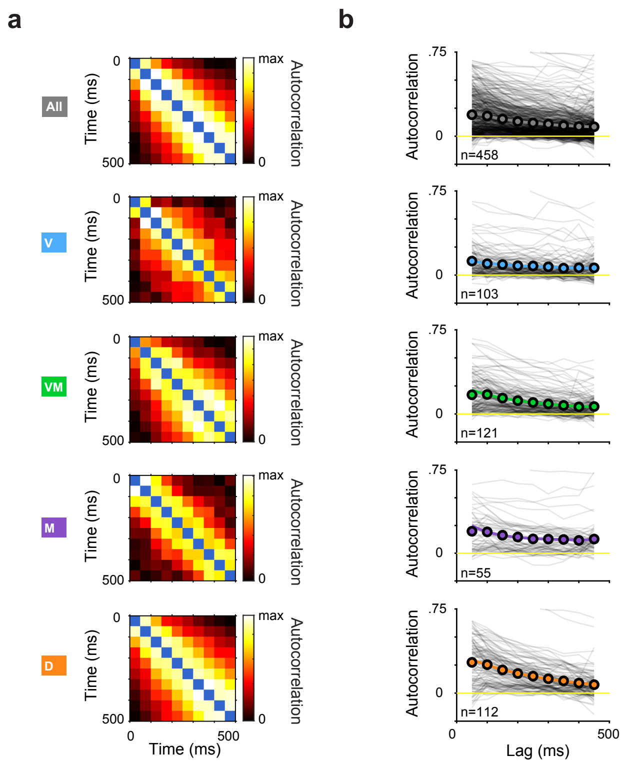

Supplementary Figure 5: Spike-count autocorrelations unwrapped in time and for single neurons, across functional class.

a Normalized spike-count autocorrelation matrices are presented for each functional class of SC neurons. Matrix elements show the mean correlation of the spike count in each time bin with the spike count in every other bin, averaged across neurons. **b** Spike-count autocorrelations are presented for individual neurons within each functional class. Colored markers indicate the population mean (identical to figure 6a). Error bars indicate 1 SEM, bootstrapped. Solid line represents the fit of an exponential decay with an offset (see Methods).
