# Supplementary material for: Correlated variability in primate superior colliculus depends on functional class
Source: Commun Biol. 2023 May 18;6:540. doi: 10.1038/s42003-023-04912-0 (PMC10195790; doi:10.1038/s42003-023-04912-0)
Supplement: Supplementary file 1 — Katz_Peer Review File [file 42003_2023_4912_MOESM1_ESM.pdf]

nature portfolio

Peer Review File

Correlated variability in primate superior colliculus depends on functional class.

Open Access This file is licensed under a Creative Commons Attribution 4.0 International License, which permits use, sharing, adaptation, distribution and reproduction in any medium or format, as long as you give appropriate credit to the original author(s) and the source, provide a link to the Creative Commons license, and indicate if changes were made. In the cases where the authors are anonymous, such as is the case for the reports of anonymous peer reviewers, author attribution should be to 'Anonymous Referee' followed by a clear attribution to the source work. The images or other third party material in this file are included in the article's Creative Commons license, unless indicated otherwise in a credit line to the material. If material is not included in the article's Creative Commons license and your intended use is not permitted by statutory regulation or exceeds the permitted use, you will need to obtain permission directly from the copyright holder. To view a copy of this license, visit http://creativecommons.org/licenses/by/4.0/.

Reviewers' comments:

Reviewer #1 (Remarks to the Author):

Summary
Katz et al. recorded simultaneously from multiple neurons in the intermediate and deep layers of the superior colliculus (SC) during conventional visually-guided ("overlapping") and memory-guided saccade tasks. Based on prior work in SC, they classified individual neurons according to their responsiveness during the visual, motor, and delay epochs of the tasks. The authors found that pairs of Delay neurons showed a pronounced increase in rsc (spike count correlation) during the delay epoch when compared to other within-class and between-class pairs of neurons. Elevated rsc in Delay pairs was true even in conditions where the saccade target was not in the putative RFs. The class of Delay neurons also exhibited the largest autocorrelations. Finally, rsc was not significantly different from zero for any classes of neurons in a subset of data collected between SC hemispheres. The authors conclude that functional class is an important consideration in the interpretation of population rsc values and that 'Delay' neuron rsc is highest due to the feedback or recurrent input required to maintain target location in working memory.

Major Comments
The paper is well written, nicely organized, and logically structured. But the results don't seem particularly surprising or insightful. I can appreciate that lumping together different classes of neurons can obfuscate the contributions of different sub-populations of neurons, and I think that is a worthwhile point to make. I find the functional definition of SC neurons to be a superficial approach to understanding cell diversity across the SC and I am unsurprised that different classes of neurons – defined solely by their firing rates across task epochs – have variable rsc values.

SC neurons were classified based on their response during four task epochs. This led to seven possible categories which were then condensed into super-classes. Although this is a classic approach in the field, it seems like a regression in methodology relative to more recent work like Khanna et. al., 2019. The authors note that Khanna and colleagues use a more continuous metric, but that it fails to capture 'Delay' like neurons and their sustained activity. This is true, however an approach where delay activity is added as an additional dimension and then neuron class can be treated as two-dimensional space in which rsc could be visualized/quantified may be more useful. Alternatively, if the authors believe distinct classes of neurons exist, a method where multiple desired metrics are added as different dimensions and then unsupervised methods are used for clustering is another potential option that is increasingly common. Something like waveform shape is one possibility along these lines. This point seems particularly important given the blurring of these functional classes in the population responses (e.g., why do Fig 1E visual neurons have a 'motor' response prior to saccade onset?).

The 'Delay' super-class is the most lenient class, combining 4 of the 7 possible categories. The largest proportion of this category are visual-delay-motor neurons (vdm). Given that the vdm neurons are responsive to every epoch it may suggest they receive the most inputs/are just more active to more of the task, making it unsurprising that 'Delay' neurons have the strongest rsc – because they just have more inputs. This could also lead to the 'Delay' neurons having the highest variability in the rsc values – which may indicate the mean rsc value reported may be less informative about these neurons. This appears to be the case given the width of the 'Delay' rsc distribution in Supplemental Figure 1 and the possible multiple modes in 'Delay' autocorrelations in Supplemental Figure 5 B (bottom panel). If this notion is correct, you would predict the next strongest rsc to be in the 'Visual-Motor' class because they are active for more epochs than the 2 most stringent classes 'Visual' or 'Motor'. It turns out that this is the case.

Peculiarities of the 'Delay' rsc over time: From Figure 2C & D, it appears that the 'Delay' class has a high rsc value during the 'foreperiod'. This correlation then decreases around the time of stimulus presentation and remains low in the visually guided task but returns to 'foreperiod' values in the memory guided task. The authors interpret this difference as working memory feedback/recurrency as driving up the correlation in the 'Delay' neurons. Could it alternatively be interpreted that visual stimulus is driving a decorrelation of these neurons? This may not be the

case given the data in Figure 4B. Nevertheless, this possibility is interesting and the strong rsc value prior to target onset is particularly intriguing in this population.

One of the main conclusions is that single value descriptions occlude an understanding of the underlying data – here they report a mean for each functional class. But are these mean values meaningful? I suspect not so much – for instance if an ideal observer was trained to classify a pair of neurons as belonging to a particular class given the rsc value – I think it would largely perform at chance except for slight proportion of the 'Delay' pairs. So although I generally agree, we should keep in mind functional classes (if they are definable in our structure) when thinking about pairwise correlations – are they really so different?

Do different functional classes have different receptive field sizes? Although it's clear they have different signal correlations it's unclear if this is due to clustering of cell types (e.g., cells with small RF clustered in same region of SC), or if the different classes have different RF size (e.g., movement fields in movement neurons smaller than visual RFs in visual neurons. If this is unknown, I think it would be helpful to mention the issue.

Minor Comments

Methods
What was the monitor refresh rate?

What were the visual parameters of the saccade targets? What color and shape was the fixation point?

Why only measure rsc to overlapping receptive fields – is it not interesting how neurons adjacent but non-overlapping relate to each other. One could imagine you may find negative correlations between these populations because of lateral interactions (e.g., Cohen, …, Schall 2010, J Neurosci).

What was the range of eccentricities and angles of targets presented across recording sessions? Where recordings across SC or focused in caudal regions? Parafoveal?

Lines 196-7: I understand that FR vs RT correlations were not significantly different across classes. Were FRs of individual classes significantly correlated with RT?

Line 580: It would be useful to include some of the settings used in KiloSort.

Line 582: Do the main results hold when using an SNR of ~1.8-2, or are there not enough neurons/pairs? An SNR of 1.5 is just above the "noise" example of 1.3 that is in the cited Kelly et al. paper.

Line 606: What was the velocity threshold?

Line 636: typo "figured"

Line 642: It says used "200 ms sliding window with 50 ms increments" but in the Figure 2 caption it says "150 ms bins in 50 ms steps".

I suggest eliminating the potential confounding of effects of different bin sizes on rsc (e.g., Huang and Lisberger 2009, J Neurophys) by keeping the analysis bin durations equivalent for the visual, delay, and movement epochs.

Discussion
This manuscript would benefit from expanded discussion on the usefulness of these findings.

Why is it significant that 'Delay' neurons have high rsc values? How does this affect encoding of information? How does this affect downstream decoding? Why might you want lower correlations

in 'Movement' neurons?

Figures
All rsc plots in the main text should be mean-matched for firing rate (as shown in the Supp Figs), especially if the firing rates are not shown alongside the rsc values.

Figure 1E – error bars for the population responses. What is the scale bar?

Figure 2 E & F – the colors are these heat maps are difficult to see. You have no negative means so the symmetrical map seems unnecessary and prohibits seeing the actual variation across bins. This comment applies to all the heat maps except supplementary figure 5.

It is challenging to interpret the data when raw firing rates are not shown. For example, in Figure 4B it is unclear if the neurons are simply not responding on trials in the out-of-RF condition.

Figure 6, Panels B and C aren't mentioned in the text?

Reviewer #2 (Remarks to the Author):

It is important to understand the structure of shared variability in neural activity because it has direct consequences for neural coding in an information theoretical sense and also reveals clues about neural circuitry. On this latter point, the question regarding how neurons with distinct functional roles are organized into circuits is of particularly great interest. The report from Katz et al tackles this question by measuring groups of neurons in superior colliculus of monkeys performing visual- or memory-guided saccades. They classified neurons into functional types depending on how their responses related to particular task epochs (visual, visuomotor, motor and delay neurons) and then quantified how shared variability (spike count correlation) differed across these groups in different task contexts. They compellingly demonstrated that the functional types of neurons did indeed differ in their shared variability (e.g., motor neurons had the weakest spike count correlations, delay neurons the strongest), and they appropriately discuss the implications of these findings in the context of the current thinking about guided saccade mechanisms and the role of shared variability more generally. The report is well-written and very professionally prepared. The science seems sound (although I have some clarifying questions), and the results and controls are persuasive. I do not have major concerns. Perhaps my greatest concern is that the authors may have inappropriately compared rsc values calculated using different count window durations (pt. 8 below). I primarily have some clarifying questions, and a few non-critical suggestions the authors might consider.

1) Please include some behavioral evidence that the animals did, in fact, perform the tasks well (e.g., percent correct behaviors).

2) Related to the previous point, were the data included for analysis conditioned on the animals' behavior? E.g., were only "correct" trials analyzed, or were all trials analyzed?

3) Still related, it would be interesting to test whether the level of correlated variability was related to the animals' behavior. For example, the authors found that delay neurons had greater correlated variability in the memory-guided saccade task than in the visual-guided saccade task (target in RF). The question then naturally arises whether that correlated variability is important for task performance. An example of a testable prediction in this case might be that spike count correlation for these neurons might be weaker on "incorrect" trials compared to on "correct" trials. An analysis such as this wouldn't be a "deal breaker" for me, but if the authors were going to do substantial revisions anyway in response to another reviewer concern, it's something they might want to consider. Alternatively, it might be the case that the animals' performance was too good to test this question (not enough incorrect trials), in which case that might be worth a mention to put my mind to rest.

4) Line 41: the presence of rsc could be consistent with common inputs, but it could also be consistent with one neuron giving input to the other neuron itself (without common input). This could be a substantial distinction, because the theoretical role of correlated variability from an information theory standpoint differs between the "common input" and "transmitter-receiver" cases. This point is discussed in: Snyder AC, Smith MA. Early steps toward understanding neuronal communication. Curr Opin Neurol. 2018 Feb;31(1):59-65. doi: 10.1097/WCO.0000000000000512. PMID: 29076880; PMCID: PMC6767631. The authors may review this article and decide whether it merits discussion in their report. I suppose I better sign this review since I'm not ashamed to plug my own stuff (as we'll see….) -Adam Snyder

5) Line 47: Another potentially relevant report the authors might consider: Snyder AC, Morais MJ, Kohn A, Smith MA. Correlations in V1 are reduced by stimulation outside the receptive field. J Neurosci. 2014 Aug 20;34(34):11222-7. doi: 10.1523/JNEUROSCI.0762-14.2014. PMID: 25143603; PMCID: PMC4138334. --Evidence for the role of feedback in rsc that is not-so-task-dependent, showing that stimulating the RF surround of V1 neurons in anesthetized monkeys decreases rsc for those neurons without necessarily an attendant change in firing rate.

6) Last one I swear: Snyder AC, Morais MJ, Smith MA. Dynamics of excitatory and inhibitory networks are differentially altered by selective attention. J Neurophysiol. 2016 Oct 1;116(4):1807-1820. doi: 10.1152/jn.00343.2016. Epub 2016 Jul 27. PMID: 27466133; PMCID: PMC5144703. – compared the attention-related change in rsc between pairs of inhibitory neurons, pairs of excitatory neurons, and mixed pairs; somewhat in the same theme as the current paper of dissecting rsc based on functional subtypes of neurons.

7) Figure 6: the pattern of intrinsic timescale strongly resembles the pattern of rsc in e.g. Figure 2H. I'm wondering how much the chosen time window duration for calculating rsc and how that "fits" with the intrinsic timescale of neurons might explain the rsc results. I.e., if rsc were counted in a 70-ms time window, might one find that motor neurons actually have stronger trial-to-trial correlations than the original analysis concluded? I guess the question is whether the rsc differences observed are "just" because of the intrinsic timescale differences, or if there is something "extra" going on. This could be addressed by e.g. repeating the analysis with different window sizes, or what Matt Smith and Adam Kohn did in their papers is calculating the integral of jitter-corrected cross-correlograms with different jitter interval durations applied (thereby destroying temporal structure on different timescales).

8) Related to the previous point, I have a reasonably strong concern about comparing rsc values calculated using different analysis window durations (shaded rectangles in Figure 2A-D, line 641 of methods). This is related to the issue that the authors correctly discussed about how rsc is biased toward zero for low rates. Well, the issue is really low *counts* (since we don't know the true underlying rate function and have to estimate that from spike counts, and the spike count estimate of rate suffers a biased quantization error near the absorbing boundary of zero). Since shorter analysis windows are going to have smaller spike counts than longer analysis windows, all else being equal (including the underlying spiking rate), rsc will be biased toward zero for shorter analysis windows. The authors need to account for this bias when comparing rsc calculated with different count window durations (e.g., Figure 2E&F, Figure 3).

9) Line 554: I'm a little confused when the authors state "Trials were distributed at a proportion of 2:1 for each pair, where targets placed in neuronal RFs were use more frequently." I think I understand this for the case where SC was recorded bilaterally (in which case there are two pairs of target locations, each pair with one location in the RF and one out), but what does this mean for the case where only one SC was recorded? What are the "pairs" of target locations in that case, and how is it that each pair has a distinct RF? Could the authors please clarify this?

We thank the Reviewers for their positive and constructive comments on our paper. The comments and suggestions were extremely helpful in improving the manuscript both on a technical and conceptual level. A point-by-point response to all Reviewer comments are presented below.

Briefly, the major changes to the manuscript are:

1. Inclusion of a new analysis which evaluates how alternative forms of neuronal classification (supervised and unsupervised), affect our results. This analysis links our "classic" method of classification with more modern forms of classification, and strengthens our main conclusion, that distinct microcircuits exist within primate SC and these exhibit different levels of correlated variability (r_{SC}). We have added a new paragraph detailing these results to the Results section, along with a new figure.
2. We have revised several sections of the text and particularly the Discussion to expand on the significance of our findings, as well as include answers to thoughtful questions made by the Reviewers.
3. We followed the Reviewers' technical recommendations—to match the duration of the delay epoch to that of the visual and movement epochs, and to enforce a more stringent inclusion criteria on our neurons—and have reanalyzed all data and regenerated all figures accordingly. Despite changes in sample size and statistics, the main effects we originally reported persist.

Below we address each of the reviewers' points and note the corresponding changes made in the revised manuscript. All references to figures in our response reference the new figure numbers.

Reviewer #1 (Remarks to the Author):

Summary
Katz et al. recorded simultaneously from multiple neurons in the intermediate and deep layers of the superior colliculus (SC) during conventional visually-guided ("overlapping") and memory-guided saccade tasks. Based on prior work in SC, they classified individual neurons according to their responsiveness during the visual, motor, and delay epochs of the tasks. The authors found that pairs of Delay neurons showed a pronounced increase in rsc (spike count correlation) during the delay epoch when compared to other within-class and between-class pairs of neurons. Elevated rsc in Delay pairs was true even in conditions where the saccade target was not in the putative RFs. The class of Delay neurons also exhibited the largest autocorrelations. Finally, rsc was not significantly different from zero for any classes of neurons in a subset of data collected between SC hemispheres. The authors conclude that functional class is an important consideration in the interpretation of population rsc values and that 'Delay' neuron rsc is highest due to the feedback or recurrent input required to maintain target location in working memory.

We thank the reviewer for their summary of our work.

Major Comments
The paper is well written, nicely organized, and logically structured. But the results don't seem particularly surprising or insightful. I can appreciate that lumping together different classes of neurons can obfuscate the contributions of different sub-populations of neurons, and I think that is a worthwhile point to make. I find the functional definition of SC neurons to be a superficial approach to understanding cell diversity across the SC and I am unsurprised that different classes of neurons – defined solely by their firing rates across task epochs – have variable rsc values.

We thank the reviewer for their positive comments and for noting that the point we make—that lumping together different classes of neurons can obfuscate the effects of spike-count correlations—is worthwhile. While this point may not be especially surprising to the reviewer, it will likely be surprising to the broader community given how common it is to encounter papers in which neurons of different classes are lumped

together. Specifically, it is common to see papers in which rsc values are reported *per area* (see for example table 1 from the seminal 2011 review by Cohen and Kohn). In our manuscript we go beyond making the point (that rsc depends on functional class) on *theoretical* grounds, and demonstrate it based on *data*, in the nonhuman primate. This is why we think our work is an important addition to the literature.

Additionally, motivated by the Reviewer's comment made below, we now also consider additional forms of functional classification, which makes the paper substantially stronger (detailed below).

SC neurons were classified based on their response during four task epochs. This led to seven possible categories which were then condensed into super-classes. Although this is a classic approach in the field, it seems like a regression in methodology relative to more recent work like Khanna et. al., 2019. The authors note that Khanna and colleagues use a more continuous metric, but that it fails to capture 'Delay' like neurons and their sustained activity. This is true, however an approach where delay activity is added as an additional dimension and then neuron class can be treated as two-dimensional space in which rsc could be visualized/quantified may be more useful. Alternatively, if the authors believe distinct classes of neurons exist, a method where multiple desired metrics are added as different dimensions and then unsupervised methods are used for clustering is another potential option that is increasingly common. Something like waveform shape is one possibility along these lines. This point seems particularly important given the blurring of these functional classes in the population responses (e.g., why do Fig 1E visual neurons have a 'motor' response prior to saccade onset?).

Great points. Let us start with the last one first.

We suspect the Reviewer was looking at the left column of Figure 1E while in fact, they should be looking at the right. The 'motor' response prior to saccade onset seen in the population response of Visual neurons is only present in the *visually guided* saccade task (left column), but not in the *memory guided saccade task* (right column), which is the task used to classify neurons into distinct classes. Thus, PSTHs on the right should map neatly onto the distinct classes and indeed, as expected by the Reviewer, there is no 'motor' response for Visual neurons during memory guided saccades. We now make this point clearer in the text (lines 102-103).

Next, we tackle the main issue raised by the Reviewer: classification strategy.

We agree that the approach taken by Khanna et al. is interesting and had, in fact, tried it out as one option among several when preparing the manuscript. We are happy to include this alternative method of classification in the paper, along with some of the other variations suggested by the Reviewer. Results of various classification approaches are presented below but briefly, we found that when the method of clustering was supervised and interpretable (e.g. binning of the visual-movement axis, like in Khanna et al. 2009), results were consistent with our own. When the method was unsupervised (e.g. k-means), the r_{SC} values across clusters were distinct (i.e. significantly different from one another, ANOVA $p << 0.01$), but it is unclear how to map these clusters to neurons in an interpretable way. Random clustering or clustering based on irrelevant epochs of the task (e.g. baseline activity) resulted in r_{SC} values that were not significantly different.

These results strengthen our paper and lend further support to our main finding, that distinct clusters of neurons exist in SC, and that these exhibit different levels of r_{SC}. We have therefore added a new section to the Results (lines 225-243 of the revised manuscript) along with a new figure (supplementary figure 3 in the revised manuscript and pasted here below).

Even though the alternative methods used in this clustering analysis are interesting, we decided to keep the primary focus of our paper on "classic" classification because it best facilitates the comparison of our results to the existing literature (which is substantial, and has almost exclusively relied on the classic classification scheme). Additionally, the classic approach maps onto specific layers and circuits in the superior colliculus, with distinct inputs and outputs. Conversely, it has yet to be determined how clusters generated via unsupervised methods map onto specific circuits. With the newly added section on classification strategy, we are now able to link between the classic approach to more recently developed classification techniques, and this may help ease the adoption of the more modern analysis techniques in the future.

Below please find the new supplementary figure. Each row in the figure corresponds to a different form of classification. To visualize the classification, we first constructed a 2D "*dPrime space*" per the Reviewer's suggestion, with the visual-movement d-prime (VM dPrime) constituting one axis and delay d-prime (Delay dPrime) constituting the other. The first row shows our original classification (termed "**classic**" classification).

(A) Panel A shows how our four classes of neurons (Visual, Visual-Movement, Movement and Delay) map onto the dPrime space (large markers indicate means). Panel A Right: r_{SC} across classes during the delay period for memory guided saccades (these are similar to data presented in Main Figure 2H. Small variations expected due to bootstrapping. Error bars are 1 SEM, bootstrapped. Gray bar reflects 95% confidence intervals on the mean of r_{SC} values for pairs drawn from a class-blind random "null" distribution, bootstrapped). We focused on r_{SC} during the delay epoch of memory guided saccades for the sake of clarity but note that results were similar for the other epochs and task conditions.

Other forms of classification (B to F):

(B) VM dPrime binning: We first considered a similar form of classification to that used in Khanna et al. 2009: binning the Visual-Movement dPrime axis into discrete bins. We chose to use 3 bins because these may roughly map onto our original V, VM and M classes, for comparison. This led to two key findings. First, different bins of neurons exhibited different levels of r_{SC} that were different than a "bin-blind" null set of pairs (gray bar in the panel). This is consistent with the main result of our manuscript. Second, bin1 (largely Movement neurons) had the lowest r_{SC}, bin2 (largely Visual-Movement neurons) an intermediate level, and bin3 (largely Visual neurons) the highest. Such an order is consistent with our own findings for Visual, Visual-Movement and Movement classes of neurons, as well as with the findings reported in Khanna et al.

(C) Delay dPrime binning: Next, we took the same binning approach and applied it on the Delay dPrime axis. We chose to use 2 bins because these may roughly map onto our original classes: those with delay activity (i.e. Delay class neurons) versus those without (the rest). We found that neurons with low delay period activity (bin1) exhibited smaller levels of r_{SC} compared to neurons with high delay period activity (bin2). This is consistent with a key finding of our manuscript, that Delay neurons exhibit higher r_{SC} during the delay period, and different from the bin-blind null set of pairs.

(D) kMeans dPrime: Following the reviewer's suggestion, we used an unsupervised clustering technique (k-means) on the data in the dPrime space. We set k to 4 to compare results to our original clustering. The technique netted 4 clusters as expected, and these loosely mapped onto the clusters obtained through our classic classification (compare to panel A). Results were somewhat consistent with those obtained by the classic approach in that the cluster corresponding to Delay neuron pairs ("Clust3") exhibited the highest r_{SC}.

(E) kMeans waveform: Again inspired by the Reviewer, here we used k-means on the mean waveform shape of each neuron. Clusters did not map onto dPrime space in an interpretable way, but they

significantly varied in r_{SC} value, lending further support to the notion that distinct clusters in SC exhibit different levels of r_{SC}, even if these aren't immediately apparent.

(F) kMeans baseline: we used k-means on neurons' activity during baseline activity (0 to 0.3 s following fixation acquisition), which is well before the target appears. In this task-irrelevant epoch, r_{SC} values did not significantly differ from one another, or from the "null" distribution of randomly selected pairs

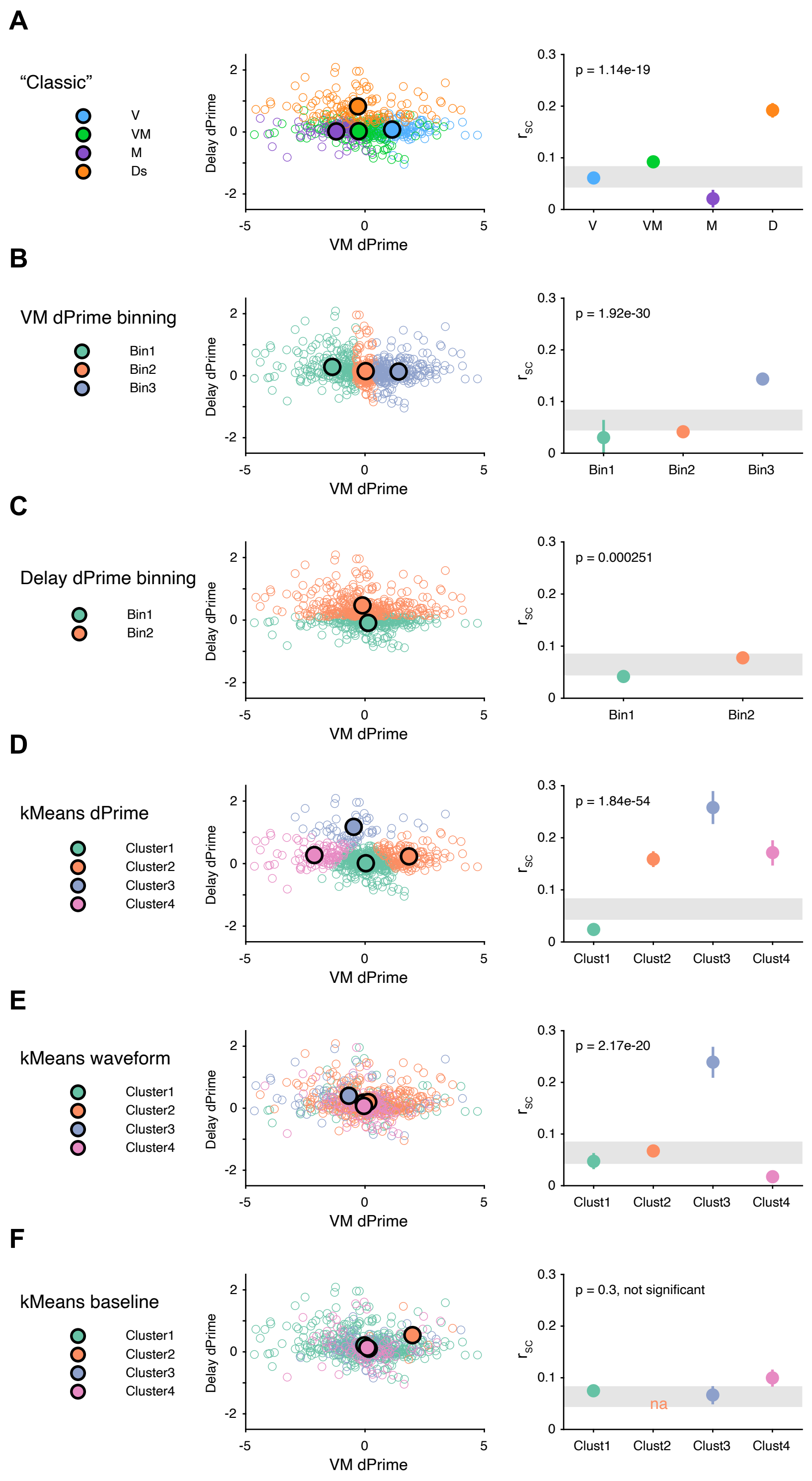

The ‘Delay’ super-class is the most lenient class, combining 4 of the 7 possible categories. The largest proportion of this category are visual-delay-motor neurons (vdm). Given that the vdm neurons are responsive to every epoch it may suggest they receive the most inputs/are just more active to more of the task, making it unsurprising that ‘Delay’ neurons have the strongest rsc – because they just have more inputs. This could also lead to the ‘Delay’ neurons having the highest variability in the rsc values – which may indicate the mean rsc value reported may be less informative about these neurons. This appears to be the case given the width of the ‘Delay’ rsc distribution in Supplemental Figure 1 and the possible multiple modes in ‘Delay’ autocorrelations in Supplemental Figure 5 B (bottom panel). If this notion is correct, you would predict the next strongest rsc to be in the ‘Visual-Motor’ class because they are active for more epochs than the 2 most stringent classes ‘Visual’ or ‘Motor’. It turns out that this is the case.

We thank the Reviewer for raising this point. There are several reasons why the higher r_{SC} in Delay class neurons cannot be explained by the interpretation that they have “the most inputs” or are “active to more of the task”. We detail these reasons below and have briefly addressed this issue in the revised manuscript (lines 759-762).

- If higher r_{SC} were due to more inputs or higher activity during more of the task, one would expect Delay class neurons to have higher r_{SC} in all task epochs. Contrary to this expectation, Delay class neurons exhibit higher r_{SC} only during the delay epoch. During the visual epoch, for example, Visual neurons have the highest r_{SC}.
- The effect of task condition on firing rates and r_{SC} shows that the strength of inputs does not explain the pattern of results. Specifically, most Delay class neurons are *less* active during memory guided saccades compared to visually guided saccades (Figure 4C) and yet their r_{SC} is higher (Figure 4B). This finding contradicts the idea that more activity during the task leads to higher r_{SC}.

Peculiarities of the ‘Delay’ rsc over time: From Figure 2C & D, it appears that the ‘Delay’ class has a high rsc value during the ‘foreperiod’. This correlation then decreases around the time of stimulus presentation and remains low in the visually guided task but returns to ‘foreperiod’ values in the memory guided task. The authors interpret this difference as working memory feedback/recurrency as driving up the correlation in the ‘Delay’ neurons. Could it alternatively be interpreted that visual stimulus is driving a decorrelation of these neurons? This may not be the case given the data in Figure 4B. Nevertheless, this possibility is interesting and the strong rsc value prior to target onset is particularly intriguing in this population.

We agree- the strong r_{SC} prior to target onset is intriguing. In fact, this finding is what motivated us to perform the autocorrelation analysis (Figure 6) where we found stronger autocorrelations in individual Delay class neurons. We do not know why r_{SC} in Delay class neurons is so high during the foreperiod but if we were to speculate, it might be linked spatial expectation. On each trial, the monkey does not know in which of 4 locations the target will appear, but it might be making predictions (i.e. guessing) on every trial. Such predictions might be made before or during the foreperiod. We do not have direct experimental evidence supporting this speculation but if it is the case that the monkey makes these spatial predictions then it is possible that Delay class neurons (that have been associated with spatial expectation) are reflecting the trial-to-trial variability in spatial prediction, leading to high levels of r_{SC} even in the foreperiod.

Is r_{SC} in Delay class neurons increased during reliance on working memory, or decreased during presentation of a visual stimulus? This is something we had previously addressed in the Discussion but perhaps not adequately. We have now rewritten the paragraph addressing this issue and point out the alternative interpretations suggested by the Reviewer more clearly (lines 510-518).

One of the main conclusions is that single value descriptions occlude an understanding of the underlying

data – here they report a mean for each functional class. But are these mean values meaningful? I suspect not so much – for instance if an ideal observer was trained to classify a pair of neurons as belonging to a particular class given the rsc value – I think it would largely perform at chance except for slight proportion of the 'Delay' pairs. So although I generally agree, we should keep in mind functional classes (if they are definable in our structure) when thinking about pairwise correlations – are they really so different?

The reviewer raises a fair criticism that we now address explicitly in our discussion of the results (lines 481-488). We do not mean to imply that the significance of spike-count correlations becomes clear once the data are subdivided into functional classes. But we do think the differences we observed across classes are meaningful, especially since the differences we found were at least as large as the changes in spike-count correlations that are implicated as significant in functions such as perceptual decision-making, learning, and selective attention. The aim of our paper is simply to illustrate that lumping all neurons in a brain region together gives a very different answer than when considering possible subclasses. We believe this point is very significant and timely, because the values based on lumping are then often deployed in models of neuronal processing that lead to general conclusions about how populations of neurons are read out for higher-brain functions.

Do different functional classes have different receptive field sizes? Although it's clear they have different signal correlations it's unclear if this is due to clustering of cell types (e.g., cells with small RF clustered in same region of SC), or if the different classes have different RF size (e.g., movement fields in movement neurons smaller than visual RFs in visual neurons. If this is unknown, I think it would be helpful to mention the issue.

Thanks for pointing this out. We now address this point to the Discussion (lines 586-597, and pasted here).

> Increased r_{SC} in neurons might also be due to RF size. Trial-to-trial variations would have larger effects on neuronal firing rates if RFs are small, and smaller effects on firing rates if RFs are large. This would translate into higher values of r_{SC} for small RFs and lower values of r_{SC} for large RFs (and negative r_{SC} values if the RFs are non-overlapping, but this scenario does not apply to our work since we only included neurons with overlapping RFs). If differences in RF size explained our results, one would expect Delay class neurons (which exhibit a relatively high r_{SC}) to have the smallest RF sizes, and Visual class neurons (which exhibit a relatively low r_{SC}) to have the largest RFs. Contrary to this prediction, previous studies have reported the opposite: that neurons in the intermediate and deep layers of the SC (where a preponderance of Delay class neurons are found) have larger RFs than those in the superficial layers (where a preponderance of Visual class neurons are found) [64,65]. Thus, differences in RF sizes across classes cannot explain the different levels of r_{SC} we measured across functional classes.

Minor Comments

Methods

What was the monitor refresh rate?

100Hz. Added to the Methods section.

What were the visual parameters of the saccade targets? What color and shape was the fixation point?

Both the fixation point and saccadic targets were 0.25° wide squares, white (48 cd/m2) on a gray background (28.5 cd/m2). These details have been added to the Methods.

Why only measure rsc to overlapping receptive fields – is it not interesting how neurons adjacent but non-

overlapping relate to each other. One could imagine you may find negative correlations between these populations because of lateral interactions (e.g., Cohen, …, Schall 2010, J Neurosci).

Absolutely agree! This question was of course addressed in our bilateral recording of SC (Figure 5) but if the Reviewer is asking about neurons within the same SC then I'm afraid we do not have the data to answer it. This is because our angle of entry into the SC is such that it maximizes the overlap of neuronal RFs (by design). There were a few neurons whose RFs do not overlap with that of the majority, but these cases were not frequent enough to provide the statistical power to address the Reviewer's question within a single SC.

What was the range of eccentricities and angles of targets presented across recording sessions? Where recordings across SC or focused in caudal regions? Parafoveal?

Most of the recordings were focused in caudal SC. Targets placed within the neuronal RFs ranged from 4° to 23° of eccentricity (median of 10°), and spanned a variety of angles. These details have been added to the Methods.

Lines 196-7: I understand that FR vs RT correlations were not significantly different across classes. Were FRs of individual classes significantly correlated with RT?

Yes (see figure below). In the Visual, Visual-Movement and Delay classes, we found an inverse relationship between firing rate (during the delay period preceding the saccade) and the saccadic reaction time, for both visually guided and memory guided saccades. An inverse correlation was also observed for the Movement class neurons, but this was not statistically significant. We find these findings interesting but because they do not relate to the main thrust of the paper, we chose not to include them.

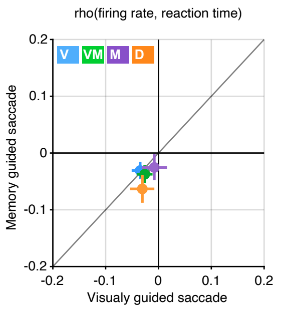

Line 580: It would be useful to include some of the settings used in KiloSort.

We have added a link to the github repository used with kiloSort2.

Line 582: Do the main results hold when using an SNR of ~1.8-2, or are there not enough neurons/pairs? An SNR of 1.5 is just above the "noise" example of 1.3 that is in the cited Kelly et al. paper.

Yes, the results hold when using more stringent thresholds of SNR, albeit with less statistical power. A modest reduction in the number of neurons leads to a substantial reduction in the number of pairs (it's an "n choose 2" problem. For example: excluding 1 of 4 simultaneously recorded Delay class neurons reduces the number of pairs by half, from 6 to 3). That said, we agree that a SNR threshold of 1.5 is on the lower end. We therefore increased our threshold to 1.8. This approach led to a loss of 6.6% in our number of neurons, 17% of Visual pairs, 26% of Visual-Movement pairs, 9% of Delay pairs, and 25% of

Movement pairs. Despite this loss in statistical power, our results remain unchanged. We thank the Reviewer for raising this point and have updated all figures and statistical tests accordingly

Line 606: What was the velocity threshold?

30°/s, updated in the Methods.

Line 636: typo "figured"

Thanks!

Line 642: It says used "200 ms sliding window with 50 ms increments" but in the Figure 2 caption it says "150 ms bins in 50 ms steps".

Thanks for catching this. The correct bin size is150. We've corrected the Methods section.

I suggest eliminating the potential confounding of effects of different bin sizes on rsc (e.g., Huang and Lisberger 2009, J Neurophys) by keeping the analysis bin durations equivalent for the visual, delay, and movement epochs.

Thanks for making this suggestion. Keeping bins equivalent facilitates an 'apples-to-apples' comparison across epochs. Reviewer 2 had also noted this (point #8), and we have now changed the duration of the delay epoch window to match the visual and movement epoch windows (namely, to a 150ms long window, from 0.65s to 0.8s following target onset, instead of the original 0.5s to 1s). All figures, text, and statistical analyses have been adjusted accordingly.

The temporal position of the 150ms long window within our original 500ms long window did not markedly change any of our results (we now note this in the Methods). Visualizing how the choice of temporal position affects all of our results would require quite a few figures. Instead, we focus on one representative figure that is in the same format as Figure 4, because Figure 4 captures two of our main results: the difference in r_{SC} across classes during the delay epoch of visually guided and memory guided saccades, and the difference between them. The figure shows that the degree of r_{SC} across classes and saccade condition is not significantly altered by varying the delay epoch position.

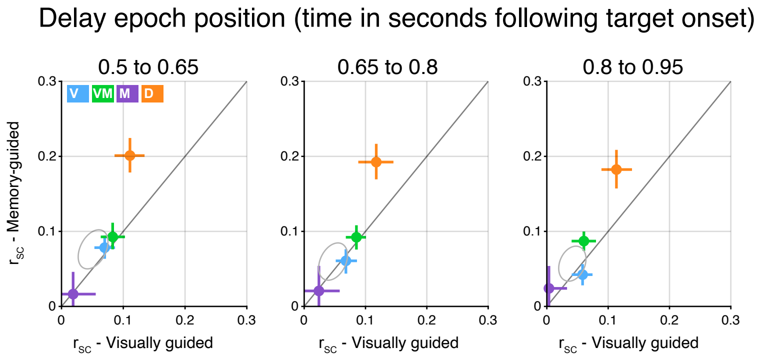

Discussion
This manuscript would benefit from expanded discussion on the usefulness of these findings.

Why is it significant that 'Delay' neurons have high rsc values? How does this affect encoding of

information? How does this affect downstream decoding? Why might you want lower correlations in 'Movement' neurons?

In the revised manuscript, our Discussion is much improved. We have expanded on several elements thanks to comments made by Reviewer 1 and 2, and these are peppered throughout the Discussion. We have now also tackled the Reviewer's question of "significance" directly. The question of significance ties into the Reviewer's question above ("when thinking about pairwise correlations – are they really so different?"), and we address the two of them jointly, in a new section of the Discussion (lines 481-488)

Figures
All rsc plots in the main text should be mean-matched for firing rate (as shown in the Supp Figs), especially if the firing rates are not shown alongside the rsc values.

The key advantage of presenting mean-matched data is that r_{SC} values are then controlled for firing rate. The disadvantage is that the data shown are no longer the raw data. We are strong proponents of showing the raw data to facilitate data comparison across labs. That said, we agree with the Reviewer that it is important to emphasize the mean matching. For our first main result—that r_{SC} depends on class—we strongly emphasize the mean matching technique in its own section with a dedicated subheading, along with references to Supplementary Figure 2A (lines 195-206). For our second main result—that r_{SC} is stronger during memory guided vs. visually guided saccades—we have now added the firing rate plots (previously Supplementary figure 4) to the main figure, such that these, as the Reviewer suggested, are presented together (new Main figure 4, and presented here below). We have updated the main text to reflect this.

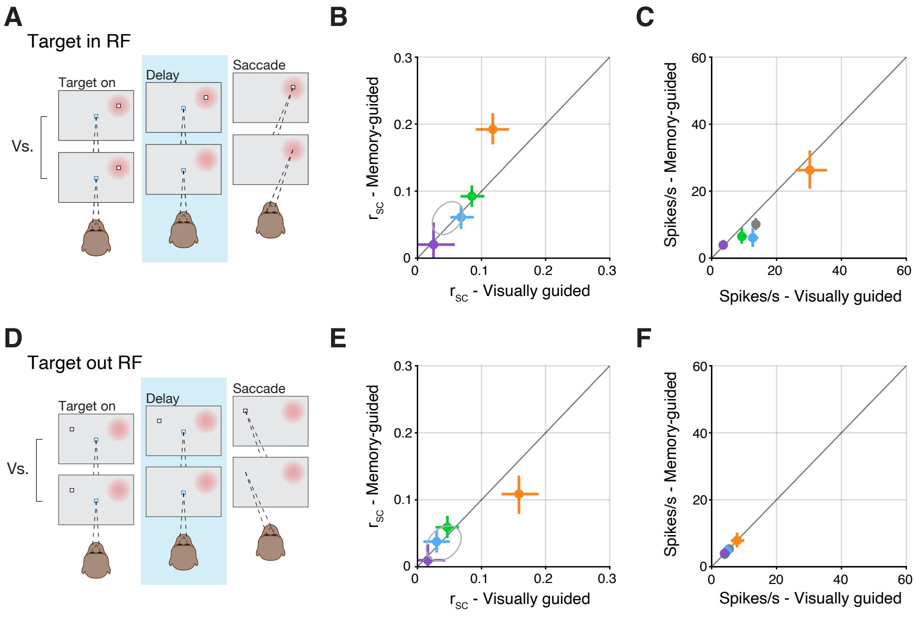

Figure 1E – error bars for the population responses. What is the scale bar?

The errorbars were so small (often smaller than the pen width of the mean) that we removed them for the sake of clarity, but we agree it is important to include them. The new version of the figure has errorbars (that are referenced in the figure caption), and the scale bar (a z-score of 1) is noted too.

Figure 2 E & F – the colors are these heat maps are difficult to see. You have no negative means so the

symmetrical map seems unnecessary and prohibits seeing the actual variation across bins. This comment applies to all the heat maps except supplementary figure 5.

As the Reviewer points out, the symmetrical is appropriate for Supplementary figure 5 (because it has a few negative means), but less so for the figures that do not. That said, we prefer having a consistent format such that interested readers could easily compare r_{SC} values across panels more readily and have therefore left the colors of the heatmap as they were.

It is challenging to interpret the data when raw firing rates are not shown. For example, in Figure 4B it is unclear if the neurons are simply not responding on trials in the out-of-RF condition.

We have now added what was previously Supplementary figure 4 to the new Main Figure 4 (in light of this Reviewer comment and the comment above) such that the figure shows firing rates alongside r_{SC} values.

Figure 6, Panels B and C aren't mentioned in the text?

Good catch! We've added text that addresses these panels in lines 407-411.

Reviewer #2 (Remarks to the Author):

It is important to understand the structure of shared variability in neural activity because it has direct consequences for neural coding in an information theoretical sense and also reveals clues about neural circuitry. On this latter point, the question regarding how neurons with distinct functional roles are organized into circuits is of particularly great interest. The report from Katz et al tackles this question by measuring groups of neurons in superior colliculus of monkeys performing visual- or memory-guided saccades. They classified neurons into functional types depending on how their responses related to particular task epochs (visual, visuomotor, motor and delay neurons) and then quantified how shared variability (spike count correlation) differed across these groups in different task contexts. They compellingly demonstrated that the functional types of neurons did indeed differ in their shared variability (e.g., motor neurons had the weakest spike count correlations,
delay neurons the strongest), and they appropriately discuss the implications of these findings in the context of the current thinking about guided saccade mechanisms and the role of shared variability more generally. The report is well-written and very professionally prepared. The science seems sound (although I have some clarifying questions), and the results and controls are persuasive. I do not have major concerns. Perhaps my greatest concern is that the authors may have inappropriately compared rsc values calculated using different count window durations (pt. 8 below). I primarily have some clarifying questions, and a few non-critical suggestions the authors might consider.

We thank the reviewer for their positive feedback!

1) Please include some behavioral evidence that the animals did, in fact, perform the tasks well (e.g., percent correct behaviors).

Good point. We have added the percent of successfully completed trials (74%) to the first paragraph of the Results (lines 82-83) and go into further details in the Methods (lines 664-665), where we define what constitutes a successfully completed trial (namely, that a saccadic end point falls within a 3° window around the saccade target).

2) Related to the previous point, were the data included for analysis conditioned on the animals' behavior? E.g., were only "correct" trials analyzed, or were all trials analyzed?

Yes, only "correct" trials were analyzed (i.e. successfully completed trials). In doing so, we minimize behavioral variability across trials such that whatever covariations we measure between pairs of neurons are considered r_{SC} (i.e. "noise correlations") as opposed to "signal correlations". We now explicitly note this in the first paragraph of the Results (lines 82-83) and in the first sentence of the "Electrophysiological analysis" section of the Methods (lines 709).

3) Still related, it would be interesting to test whether the level of correlated variability was related to the animals' behavior. For example, the authors found that delay neurons had greater correlated variability in the memory-guided saccade task than in the visual-guided saccade task (target in RF). The question then naturally arises whether that correlated variability is important for task performance. An example of a testable prediction in this case might be that spike count correlation for these neurons might be weaker on "incorrect" trials compared to on "correct" trials. An analysis such as this wouldn't be a "deal breaker" for me, but if the authors were going to do substantial revisions anyway in response to another reviewer concern, it's something they might want to consider. Alternatively, it might be the case that the animals' performance was too good to test this question (not enough incorrect trials), in which case that might be worth a mention to put my mind to rest.

This is a wonderful question, and per the Reviewer's request, have addressed it in lines 218-220. In fact, we have considered this question previously, but found no relationship between behavior and correlated variability. We share our method with the Reviewer: we split our behavioral dataset into three thirds: trials with high saccadic accuracy (i.e. small end point error, up to 0.75° away from target location), medium (0.75-1.5°), and low (1.5-3°). All of these are considered "correct" trials, but the end point error magnitude is a proxy for "*how* correct" a trial is). We performed a similar analysis on trials with either low or high saccadic reaction times (RT).

The figure below is in the same format as our Main Figure 4, because Figure 4 captures two of our main results: the difference in r_{SC} across classes during the delay epoch of visually guided and memory guided saccades, and the difference between them. The figure shows that r_{SC} did not vary with saccadic accuracy (top row) or RT (bottom). This result may indicate that r_{SC} during the delay epoch simply does not correlate with behavior. Alternatively, it might be the case and the Reviewer is correct, that because the task allows for saccadic errors up to 4 degrees of visual angle, and does not reward subjects for hastened responses, performance was "too good to test this question". A task design where the subject is motivated to perform more accurately (e.g., higher accuracy leads to higher reward), for example, might be better suited to address this question directly.

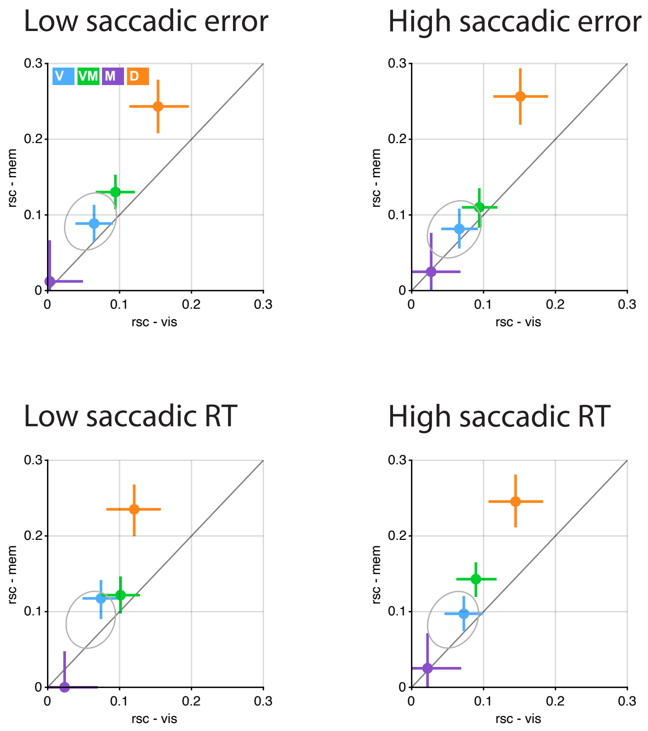

4) Line 41: the presence of rsc could be consistent with common inputs, but it could also be consistent with one neuron giving input to the other neuron itself (without common input). This could be a substantial distinction, because the theoretical role of correlated variability from an information theory standpoint differs between the "common input" and "transmitter-receiver" cases. This point is discussed in: Snyder AC, Smith MA. Early steps toward understanding neuronal communication. Curr Opin Neurol. 2018 Feb;31(1):59-65. doi: 10.1097/WCO.0000000000000512. PMID: 29076880; PMCID: PMC6767631. The authors may review this article and decide whether it merits discussion in their report. I suppose I better sign this review since I'm not ashamed to plug my own stuff (as we'll see….) -Adam Snyder

Hi Adam, thanks for bringing this to our attention. The transmitter-receiver architecture is important to consider and is consistent with our interpretation of the increased levels of r_{SC} during periods that rely on working memory. We have addressed this architecture as another possibility in our Discussion (lines 532-533).

5) Line 47: Another potentially relevant report the authors might consider: Snyder AC, Morais MJ, Kohn A, Smith MA. Correlations in V1 are reduced by stimulation outside the receptive field. J Neurosci. 2014 Aug 20;34(34):11222-7. doi: 10.1523/JNEUROSCI.0762-14.2014. PMID: 25143603; PMCID: PMC4138334. --Evidence for the role of feedback in rsc that is not-so-task-dependent, showing that stimulating the RF surround of V1 neurons in anesthetized monkeys decreases rsc for those neurons without necessarily an attendant change in firing rate.

Thanks for the reference. We have considered how the results from Snyder et al. 2014 map onto our own (and in particular in the context of the "out-RF" results, Figure 4, bottom row) but failed to find a direct link because the normalization mechanisms proposed in the Reviewer's paper are *within* a hemifield (surround suppression of the RF) whereas our effects are *between* hemifields. Additionally, a key feature of our results is that they are task-dependent, whereas those in Synder et al. were obtained in the anesthesia prep and are task-independent.

6) Last one I swear: Snyder AC, Morais MJ, Smith MA. Dynamics of excitatory and inhibitory networks are differentially altered by selective attention. J Neurophysiol. 2016 Oct 1;116(4):1807-1820. doi: 10.1152/jn.00343.2016. Epub 2016 Jul 27. PMID: 27466133; PMCID: PMC5144703. –compared the attention-related change in rsc between pairs of inhibitory neurons, pairs of excitatory neurons, and mixed pairs; somewhat in the same theme as the current paper of dissecting rsc based on functional subtypes of neurons.

Thanks, this a great reference that is well within our theme. We have added it to the Discussion section (lines 501-503).

7) Figure 6: the pattern of intrinsic timescale strongly resembles the pattern of rsc in e.g. Figure 2H. I'm wondering how much the chosen time window duration for calculating rsc and how that "fits" with the intrinsic timescale of neurons might explain the rsc results. I.e., if rsc were counted in a 70-ms time window, might one find that motor neurons actually have stronger trial-to-trial correlations than the original analysis concluded? I guess the question is whether the rsc differences observed are "just" because of the intrinsic timescale differences, or if there is something "extra" going on. This could be addressed by e.g. repeating the analysis with different window sizes, or what Matt Smith and Adam Kohn did in their papers is calculating the integral of jitter-corrected cross-correlograms with different jitter interval durations applied (thereby destroying temporal structure on different timescales).

This is an interesting line of thought and we've now analyzed the data with a differently sized window per the Reviewer's suggestion. The figure below is again in a similar format to Main Figure 4, but here, the epoch of interest is the movement epoch (and not the delay). We compared our standard window (150ms around the saccade) to a 100ms* window (25ms before up to 75 ms after). (*we chose 100ms and not 70 because any window smaller than 100ms could lead to artifactual measurements of r_{SC}, see Figure 3c of the Cohen and Kohn 2011 review). In our comparison, we find no difference in the r_{SC} values of Movement class neurons across window sizes. This is likely because our standard window size (150 ms) is sufficiently short to capture autocorrelation effects with time constants as small as 85ms (the Movement class neurons) given that 85ms is the time at which the autocorrelation reaches 1/e of its initial value, not the time at which is ceases to persist.

Movement epoch duration (time in seconds relative to saccade)

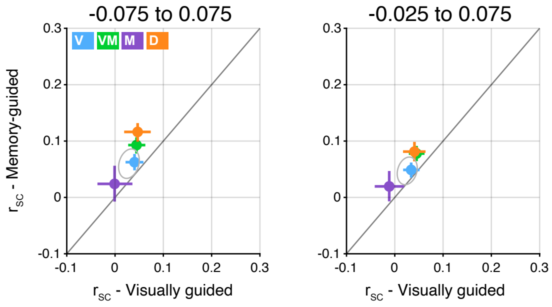

8) Related to the previous point, I have a reasonably strong concern about comparing rsc values calculated using different analysis window durations (shaded rectangles in Figure 2A-D, line 641 of methods). This is related to the issue that the authors correctly discussed about how rsc is biased toward zero for low rates. Well, the issue is really low *counts* (since we don't know the true underlying rate function and have to estimate that from spike counts, and the spike count estimate of rate suffers a biased quantization error near the absorbing boundary of zero). Since shorter analysis windows are going to have smaller spike counts than longer analysis windows, all else being equal (including the underlying spiking rate), rsc will be biased toward zero for shorter analysis windows. The authors need to account for this bias when comparing rsc calculated with different count window durations (e.g., Figure 2E&F, Figure 3).

Thanks for making this suggestion. Reviewer 1 had also noted this concern and we have reanalyzed our data accordingly. We paste our response to Reviewer 1 below:

> Thanks for making this suggestion. Keeping bins equivalent facilitates an 'apples-to-apples' comparison across epochs. Reviewer 2 had also noted this (point #8), and we have now changed the duration of the delay epoch window to match the visual and movement epoch windows (namely, to a 150ms long window, from 0.65s to 0.8s following target onset, instead of the original 0.5s to 1s). All figures, text, and statistical analyses have been adjusted accordingly.
>
> The temporal position of the 150ms long window within our original 500ms long window did not markedly change any of our results (we now note this in the Methods). Visualizing how the choice of temporal position affects all of our results will require quite a few figures. Instead, we focus on one representative figure that is in the same format as our Main Figure 4A, because Figure 4A captures two of our main results: the difference in r_{SC} across classes during the delay epoch of visually guided and memory guided saccades, and the difference between them. The figure shows that the degree of r_{SC} across classes and saccade condition is only minimally changed by varying the delay epoch position.

Delay epoch position (time in seconds following target onset)

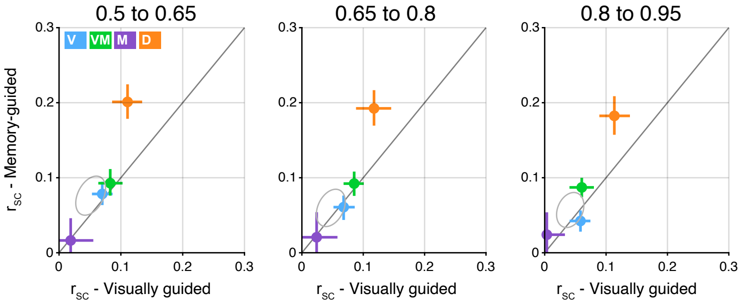

9) Line 554: I'm a little confused when the authors state "Trials were distributed at a proportion of 2:1 for each pair, where targets placed in neuronal RFs were use more frequently." I think I understand this for the case where SC was recorded bilaterally (in which case there are two pairs of target locations, each pair with one location in the RF and one out), but what does this mean for the case where only one SC was recorded? What are the "pairs" of target locations in that case, and how is it that each pair has a distinct RF? Could the authors please clarify this?

We understand the confusion. We have rewritten that section of the Methods to clarify (lines 670-681). Briefly, the task was designed with four candidate locations for a target on any given trial (two on each hemifield), regardless to whether we recorded from one SC, both SCs, or neither SC. We always had an equal number of trials to either hemifield (as opposed to focusing only on the recorded hemifield when only 1 SC was recorded from) to keep the task similar across conditions, and to reduce spatial expectation to the extent possible.

REVIEWERS' COMMENTS:

Reviewer #1 (Remarks to the Author):

I appreciate the amount and quality of work that the authors have put into their revisions. The previous issues raised were sufficiently addressed, and there are enough details in the manuscript to evaluate the claims.

Reviewer #2 (Remarks to the Author):

The authors have satisfied my concerns. Great work!
